# Ignorance is bliss? Information and risk on crowdfunding platforms

**DOI:** 10.1371/journal.pone.0286876

**Published:** 2023-06-16

**Authors:** Chiara D’Arcangelo, Azzurra Morreale, Luigi Mittone, Mikael Collan

**Affiliations:** 1 Department of Business Economics, University of Chieti-Pescara, Pescara, Italy; 2 LUT Business School, LUT University, Lappeenranta, Finland; 3 Department of Economics and Management, University of Trento, Trento, Italy; 4 VATT Institute for Economic Research, Helsinki, Finland; Privatuniversität Schloss Seeburg: Privatuniversitat Schloss Seeburg, AUSTRIA

## Abstract

This research examines the determinants of project success on crowdfunding platforms within a competitive context. We focus on the specific horizontal attributes of the project–attributes that do not affect the project returns but over which investors may have heterogeneous preferences–and on the project returns’ risk level. We run a laboratory experiment with several set-ups, where multiple projects compete for funding simultaneously and where potential investors operate in a quasi-continuous time. We find the horizontal attributes’ information affects project selection, while the risk level of the project returns affects the amount of collected funding.

## Introduction

Crowdfunding (CF) is a form of financing that relies on gathering small amounts of funding from many individuals–the ‘crowd’. Over the past few years, taking advantage of the opportunities granted by the Internet, the number of crowdfunded investments and funding volume has dramatically increased, reaching billions. This number is expected to grow [1]. CF platforms typically host hundreds (sometimes thousands) of projects at the same time so that, even if the crowd is motivated by different aims, and, despite some projects having a peculiar design that might attract public interest, most of the projects have to compete against other, similar projects, simultaneously looking for funding [2]. Because there is no ‘optimal’ project here, investors must use the available information to infer which project is more likely to be selected by other investors. Thus, it is important for the project creators to wisely choose the information disclosed to potential investors to attract their attention. Indeed, as [3] points out, CF is a noisy environment in which several signals compete to get investors’ attention. An interesting paper that copes with the empirical analysis of a complex set of information crowdfunding environment using a *Fixed Estimation Panel Data Model* is [4]. This paper underlines, among the other results, the importance of the bakers’ herding behavior in the preliminary stages of a crowdfunding campaign. In this context, certain bundles of signals, such as the entrepreneur’s education [5], or the entrepreneur’s past CF experiences [6, 7], might complement the information about the characteristics of the project. Accordingly, several contributions have explained which characteristics of the project and the entrepreneur contribute to successful fundraising and other information [4, 8–11]. We know less about how such signals interact with information provided over the risk level of project returns. In fact, in real-world CF, even if a project is successfully funded, it is still subject to the risk of not generating the promised returns [12], which might result from market acceptance [13], delayed compensations or even project failure [14]. For instance, about 75% of successfully funded projects on Kickstarter were delivered with a substantial delay, and around 4% were never delivered [15]. Projects can fail to succeed in the market [13] or to acquire the needed successive rounds of funding from professional investors, such as venture capitalists [16]. Therefore, information about the project (in terms of the project’s finance and risk) is relevant [9] because it provides an understanding of the expected return of a project [8, 17]. Accordingly, industry experts managing CF platforms (such as Kickstarter) recognize that backers face such risk, and they implemented ways to give more information to investors [15]. For instance, in 2012, Kickstarter introduced a ‘*Risks and Challenges’* section to campaign pages, where project designers were required to indicate possible risks and solutions [18]. The academic literature acknowledges that potential backers face market uncertainty and highlights the importance of risk disclosure [19, 20]. Despite both practitioners and academic scholars highlighted the importance of disclosing risk, empirical evidence on how the diverse levels of risk might affect CF decisions is still scarce [18]. In the current study, we aim to fill this gap and investigate, theoretically and empirically, about two specific aspects of a CF campaign: the provision of information about some specific horizontal attributes of a project and the risk level of the project’s returns. The horizontal attributes refer to characteristics of the projects and of the projects’ designers–i.e., the personal characteristics of the entrepreneur who is launching the project. These attributes, as well as other informational contents, do not directly affect the project’s returns but can nevertheless influence the project’s success. More specifically we argue that the effect of introducing information about horizontal attributes when projects’ returns are risky is not trivial. On the one hand, if this information drives the backers’ attention towards a project that is also preferred according to their risk attitudes, it can increase contributions to the winning project. On the other hand, if the information about the horizontal attributes drives the backers’ attention towards a project that is not preferred according to their risk attitudes, it can have the opposite effect, thereby decreasing contributions to the winning project. Indeed, in this case, it may be more difficult for backers to identify an option as preferred because they might face a trade-off between their risk preferences and their beliefs about the expected success of a salient project, whenever they do not match. Shedding light on how these two factors (information about horizontal attributes and risk) interact to determine coordination and contributions in a multi-projects setting is the main contribution of our study.

To analyze this issue from a theoretical perspective, drawing on [21, 22], we model the CF process as a coordination game in which an investor gets a positive return from a project only if the same project is chosen by many other investors. Based on this theoretical framework, we conduct a laboratory experiment to investigate investors’ behaviour. Despite its importance, there is scarce evidence, at the micro-micro level of the individual lender, about the causal relationship between the selection process and the following decisions of investment in CF projects [23]. Our experimental design allows us–for the first time to the best of our knowledge–to isolate and disentangle the effect of the risk level of the project returns from the effect of information about some specific horizontal attributes of the project. Importantly, we developed a laboratory-based CF setting that allows us to better simulate a real-world CF process. First, in our setting participants can make asynchronous contributions over a quasi-continuous time, i.e., over 10 periods (rounds),–being informed, in each period after the first one, of how much each project was able to collect up to that period. Second, our experimental setting accommodates multiple projects and various CF campaign designs, including scenarios where only one project can win the competition (i.e., be financed) and others where more projects can be financed. This enables us to study investment behavior in both competitive and less competitive environments.

Hence, we make three main contributions. First, our study builds upon recent CF literature that uses field experiments to examine the effects of *signals*, which are attributes of the projects (or of the projects’ designers) that can reduce information asymmetry for investors [13, 23]. We extend this research by explicitly accounting for a major exogenous risk that affects CF projects even after successful funding. By disentangling the role of risk from the one of horizontal attributes, we explore how each factor’s effectiveness is affected when both are present.

Second, while there have been efforts to study the micro-foundations of CF through laboratory experiments [2, 24–27], most of the previous studies focus on donation-based CF, and model the CF funding process as a public goods game, where contributors get a benefit from a successfully funded project regardless of their participation In our research, we model the CF process as a dynamic coordination game with a threshold, shedding light on investors’ behavior in contexts where they only receive a monetary return if they contribute. This approach captures the dynamics of various types of CF, including Equity-based CF or Royalty-based CF. Moreover, our experiment simulates real-life CF platforms by allowing for contributions to multiple projects in a quasi-continuous environment.

Third, our study also contributes to the growing literature on CF by providing insights into the interplay between horizontal attributes and exogenous risk when multiple projects compete for limited funding. While some CF campaigns attract public interest, many projects are indistinguishable from one another [2]. Given investors’ limited resources, funding one project may mean less funding for similar campaigns, making CF a zero-sum game [28]. However, previous research has mainly focused on fundraising within individual campaigns, with little attention paid to cross-campaign dynamics [29]. Typically, cross-campaign dynamics in CF have only been examined through a control measure of the number of concurrent campaigns [30]. This is due to the difficulty of using observational data to determine whether users visit multiple campaigns before contributing to a specific project. Our unique experimental design enables us to investigate this phenomenon and observe the causal effect of horizontal attributes and risk on investor decision-making. We consider different levels of competition among projects, which reflect real-world situations on CF platforms.

Our results shed light on the crucial importance of considering and isolating the risk of the project returns from the effect of information about some specific horizontal attributes of the project. We show that: i) when participants are informed only about the project’s horizontal attributes and the projects’ returns are certain, they are more likely to invest in a project with a specific vector of attributes; ii) when the projects’ returns are risky, but no information about the characteristics of the projects is provided, the participants are more likely to choose the lowest-risk projects, but contributions to the funded project were significantly lower than in the previous case (i); iii) finally when returns involve risk and information about horizontal attributes is available, our findings suggest that horizontal attributes are the primary factor in determining which project is successful. However, risk becomes the dominant factor in determining the amount of investment in the winning project. Indeed, in such a case, the successful projects collected the lowest amount of funding and more participants decided not to invest at all.

Our research has important practical implications for project creators and for companies seeking funding via CF platforms and companies that provide and manage CF services. Our findings shed light on the factors that influence investor decision-making in CF, particularly in the context of multiple projects competing for funding. Despite the many advantages associated with CF, some entrepreneurs may still be reluctant to take part in CF campaigns due to the costs involved [14]. Our research emphasizes the critical importance of horizontal qualities in predicting project success and offers project founders useful guidance on what types of information to provide to investors and how to design their campaigns to attract funding.

Moreover, our findings imply that project developers should carefully evaluate the degree of risk associated with their project. We advise project creators that if they cannot disclose specific horizontal attributes, investors are more likely to invest in low-risk projects. Therefore, project creators should consider incorporating low-risk attributes into their projects and communicate them to investors to increase their chances of success. Finally, our findings suggest that, even if the presence of risk can decrease total contributions, providing the right piece of information about the horizontal attributes of a project might be crucial to help investors coordinate toward the same project.

Overall, our findings can inform the designers of CF campaigns and platforms, potentially increasing their effectiveness and helping entrepreneurs and creators raise the necessary capital for their projects.

## Experimental approach to CF

Since the publication of the work by [31], a wide body of experimental work has examined how different funding mechanisms affect investors’ contributions [32–35], mostly focusing on settings in which only one project is available for funding.

Recent literature [2, 24–26] has examined coordination and investors’ contributions when multiple projects (public goods) with similar or indistinguishable characteristics are available for funding. The main finding of this literature is that coordination becomes more difficult when participants face multiple options, that is there is a considerable drop in provision rates when multiple projects are available for funding [26]. However, this effect is mitigated when one of the options becomes salient: ensuring higher returns—as shown by [2]—or seeding money to a project—as shown by [25]—can be effective in increasing contributions. The experiments of [2, 24] requested participants to make decisions in a static environment (i.e. simultaneous decisions) and, thus, cannot capture the dynamic nature of CF campaigns: typically, a CF campaign collects contributions over a certain period, and investors are continuously informed about the amount of funding already collected. This aspect is considered by [25, 26]. Similarly, we gave the participants the possibility to decide in a dynamic environment, that is, in a quasi-continuous environment where participants made fully informed decisions over 10 rounds.

While the above-mentioned papers provide relevant insights on coordination toward multiple projects, we differ in that we consider information regarding both the projects and the projects’ designers. Indeed, drawing on signaling theory [36, 37], numerous studies on CF highlight that showing the attributes concerning project designers or the project plays a crucial role in the selection of the winning projects and in determining the amount of collected funding [8–11]. Providing such information can make a project salient with respect to other projects (in the spirit of [38]) and can increase contributions towards that project [39, 40] because it can indirectly signal the quality of the project, thus reducing the information asymmetry faced by CF investors.

In this respect, we are more closely related to the experimental study of [23] who adopted a randomized field experiment to examine the effect of several types of information, such as the popularity of the CF campaign, the presence of a leading investor, and the project’s creators’ background, finding that investors respond more strongly to the information regarding the human capital (i.e., the creators) of the project. Using a similar experimental design, [13] complements the work of [23] by considering a fourth signal, that is, information related to product characteristics. Her main findings show that investment-related information is effective only when combined with product-related information. These results highlight the importance of considering different signals to estimate the conditional probability that a project will succeed.

However, little is known about how individuals react to the information regarding the riskiness of the project (and different levels of risks), that is how providing a precise signal of risk (i.e., an objective probability of success) affects subjects’ funding decisions. To the best of our knowledge, the only work dealing with this issue is the work by [18], who conducted an online experiment to examine the effect of risk disclosure on participants’ hypothetical funding decisions. The Authors report that projects’ risk disclosure and risk level have different effects depending on the complexity of the project. In their study, the participants are provided with qualitative measures of risk. The authors use sentences like: ‘*We’re still working on changes to provide you with the best 3-D printing experience you’ve ever known*’ or ‘*We’re still working on minor changes to provide you with the best 3-D printing experience you’ve ever known*. *By January*, *we will have it thoroughly tested’* ([18] p. 18) to communicate a situation of ‘high risk’, or ‘low risk’, respectively. We believe this leaves room for speculation about the subjective perceptions of the diverse levels of risk.Our paper contributes to this literature, by examining coordination and contributing behavior when multiple projects are available for funding under a setting that simultaneously: 1) allows for contributions in a quasi-continuous time, and 2) encompasses two aspects of a CF campaign: the information about the horizontal attributes of the project and its designer, and the degree of risk over the project returns.

Moreover, we consider the role played by different degrees of competition among projects, manipulating the number of potential winning projects and the range of the investment choice alternatives allowed to participants. We conducted three studies to capture important characteristics of real-life CF. In Study 1, investors could only invest in one project, and a maximum of one project could reach the funding threshold. Study 2 allowed investors to distribute their initial endowment among the available projects, while Study 3 allowed multiple projects to reach the funding threshold. The experimental setups of Study 1 and Study 2 reflect the situations present in small CF platforms with a limited number of projects and investors, such as Fundu.fi, Estateguru.co, and groundfunding.fi, as well as investment- or loan-based CF platforms like Lending Club, where cross-campaign competition and serial contributions are common. The type of platforms reflected in Study 3, such as Kickstarter.com and Indiegogo.com, represents large players in the field of venture capital financing.

Finally, our experimental setup is distinct from other related studies, as it allowed participants to replicate the investment decision making process three times with different sets of competing projects, providing a unique opportunity to study the role of experience in investment decision-making. This is especially important because previous research has suggested that investors with varying levels of experience tend to evaluate CF projects differently [41]. Thus, our study takes a more nuanced approach to understanding how investor experience can impact decision-making in CF. By allowing participants to repeat the decision-making process, we can more accurately capture how experienced investors may evaluate projects differently from novice investors. These findings can help inform the designers of CF platforms to better meet the needs of different types of investors, ultimately improving the chances of project success.

## Theoretical framework and model set up

We aim to assess whether, and how, the presence of information regarding certain horizontal attributes and/or the presence of risk, affect the project’s funding success. To analyze the problem from a theoretical perspective, we follow [21, 22], and we model the CF process as a coordination game, one in which an investor will get a positive return from a project only if the same project is chosen by many other investors. A game like this is characterized by the so-called equilibrium selection problem [42] which, in our setting, translates to a “project selection problem”. In the following, we study this problem by first considering the case of identical projects, and then analyzing the impact of our variables of interest, that is, the information about horizontal attributes and the risk level of projects’ returns.

### Standard and behavioral predictions: A basic theoretical model

We consider a game played among *N*≥2 potential investors of *J*≥2 competing projects. Each project has the same funding threshold, *k*, and the same deadline, *T*, so that it will generate a return for its investors only if it collects enough funding to reach the threshold before the deadline–in this case, we call it a “winning project”. Thus, differently from previous models, we consider a dynamic setting, in which players have several periods available to make their decisions. Specifically, each player has an initial endowment equal to *e*, which he can spend over *T* periods, knowing, in every period after the first one, the amount of funding collected by each project up to that period.

The decision of player *i* in period *t* regarding project *j* is $x_{ij}^{t}=b_{ij}^{t}e$, with $0\leq b_{ij}^{t}\leq 1$ and $\sum_{j,t}b_{ij}^{t}\leq 1$. For each project, if, in period *T*, $\sum_{i,t}x_{ij}^{t}\geq k$, so that *j* is a winning project, player *i* will get a return equal to $\sum_{t}b_{ij}^{t}R$; otherwise, he will get a partial refund equal to $\sum_{t}b_{ij}^{t}c$, with *c*<*e*<*R*.

#### Identical projects

Consider first the case of identical projects, so the only information that a player has is the amount of funding previously collected by each project. A strategy must then specify a decision for every project *j*, every period *t*, and, if *t*>1, any possible allocation. This game has multiple equilibria, which can be classified into 4 types (see S1 Appendix for a detailed analysis):

- *not investing*: $b_{ij}^{t}=0$ for all players, all projects, and all periods;
- *full investment*: $\sum_{j,t}b_{ij}^{t}=1$. All players invest their full endowment, and all investments go to winning projects, no matter the order in which decisions are taken;
- *invest at last*: players wait to invest until the very last period, and then they all invest, using a mixed strategy in which (at least) two projects are chosen with the same probability;
- *mixing*: players use a mixed strategy also before the last period of the game, investing with a relatively low probability, with little investment at the beginning of the game. When the collected funding for some project reaches a critical value, all investments go towards that project.

Thus, in all equilibria in which there is a winning project, players invest their full endowment. Moreover, as projects are identical, each project has the same probability to be successfully funded.

STP1a (Standard theoretical prediction 1a): With identical projects, in all equilibria in which there is a winning project, players invest their full endowments.

STP2b. With identical projects, each project has the same probability to be funded.

#### Horizontal attributes

Consider now the case in which potential investors have access to information regarding certain projects’ horizontal attributes. This information can be modeled as a signal that might reduce information asymmetries without affecting the project’s returns. As an example, consider the case of two projects, A and B, and two possible signals, *m*_1_ and *m*_2_. To each project is randomly assigned a signal, so that two combinations are possible: (*Am*_1_, *Bm*_2_) or (*Am*_2_, *Bm*_1_). A strategy must then specify a decision for each possible combination. For example, the strategy profile in which all players “follow” the signal *m*_1_, regardless of which project the signal is attached to, is an equilibrium of the game. The presence of information expands the equilibrium set, and it might be more difficult for players to coordinate on the same project. Nonetheless, if one of the signals, say *m*_1_, can make a project focal, coordination should be easier and, more importantly, projects associated with *m*_1_ should be more likely to be successfully funded. Of course, in this way we are introducing the assumption that players can distinguish between “focal” and “non-focal” projects, assumption that seems well supported by the CF literature [28, 43, 44] that refers to “behavioral factors” that can support the focalizing process among bakers. Thus, if a specific combination of attributes can make a project salient with respect to others, we should observe winning projects sharing the same characteristics and, if this is the case, winning projects should also be able to collect more funding, compared with the case of identical projects.

*BP1a* (*Behavioral Prediction 1a)*: *winning projects share the same horizontal attributes*.

BP1b: the amount collected by winning projects is higher when horizontal attributes are provided to the players than in situations where the investors cannot distinguish among projects.

#### Risk

While players’ returns are not affected by the presence of information over horizontal attributes, this is not the case when projects’ returns are uncertain. In fact, players’ risk preferences might now affect the amount of collected funding. To see this, consider again the case of two projects, A and B, and assume that to each project is associated a lottery (*L*_*j*_, *p*_*j*_): if project *j* reaches the threshold, it will generate the return *L*_*j*_ with probability *p*_*j*_, while with probability (1−*p*_*j*_), the project fails, and its investors get nothing. Assume also that *L*_*j*_*p*_*j*_ = *R* for all *j*, so that a risk neutral player would be indifferent between the two projects, and 1>*p*_*A*_≫*p*_*B*_>0 so that project A is relatively safer than project B. If these are the only available projects, risk adverse players might choose to invest in A, but not in B. If they are sufficiently risk averse, they could choose not to invest at all, even if a project already reached the threshold. Thus, with risky returns, a low-risk winning project should collect more funding than a higher-risk one, as it also collects funding from those risk-averse players who would not invest in high-risk projects. Moreover, we should expect a lower amount of funding collected by the winning projects, if compared with the case with certain returns.

STP2a: Low risk projects collect more funding than high risk ones.

*STP2b*: *Winning projects collect more funding with certain*, *rather than uncertain*, *returns*.

#### Horizontal attributes and risk combined

To complete the analysis, we consider the case in which the information is available, and projects returns are risky. That is, to each project are associated both a signal about horizontal attributes, and a lottery to be run in the case it is successfully funded. On the one hand, if signals can drive the players’ attention towards a project that is also preferred according to their risk attitudes, it can increase contributions to the winning project. On the other hand, if signals drive players’ attention towards a project that is not preferred according to their risk attitudes, it can have the opposite effect, decreasing contributions to the winning project, as players might face a trade-off between their risk preferences and their beliefs about the expected success of a salient project, whenever they do not match. A purely speculative hypothesis is that, when both information and risk are present, it might be more difficult for players to identify a single option as salient, and this might lead them to diversify investment, and even discourage contributing to the first place.

BP2: when horizontal attributes are provided and returns are risky, the amount collected by winning projects is lower if compared with situations where returns are certain, or where returns are risky, but the information is not provided.

#### Competition

A further aspect that we want to analyze is the degree of competition among projects, as CF platforms often vary in the number of projects simultaneously looking for funding, and in the number of users (that is, of possible investors). With this aim, we consider three different funding mechanisms, which we analyze in three studies. In Study 1 and Study 2, the value of the funding threshold is such that there can be, at most, one winning project. The situations reflected in these two studies have real-world counterparts in small CF platforms with a limited number of projects and investors present at any time. Several such platforms operate, for example, in the Nordic and the Baltic countries and are exemplified by the likes of Fundu.fi, Estateguru.co and groundfunding.fi. The only difference between the two is that, while in Study 2 investors can divide their endowment into different investment decisions, in Study 1 the decision is binary, so that one can either invest the full endowment, or not invest at all, an experimental feature that was shown to be linked with an increased likelihood to make optimal choices [45]. In Study 3 instead the value of the funding threshold is such that there can be more than one winning project, decreasing, in a sense, the competition among the projects. The type of platforms reflected by this Study exists internationally, and the best-known of these, such as Kickstarter.com and Indiegogo.com, have grown to become large players in the field of venture capital financing.

From a theoretical point of view, moving from Study 1 to Study 3, the number of equilibria increases, making the project-selection problem even more severe. Therefore, and perhaps counterintuitively, reducing the degree of competition–given the specific definition of competition here assumed–might actually decrease the amount of funding collected by winning projects:

*BP3*: *Moving from Study 1 to Study 3*, *the amount collected by winning projects decreases*.

#### Experience

The last dimension that we investigate regards the effects of acquiring experience during time across many repetitions of the investment choices. This decision is supported by the literature that investors with different experiences react to and evaluate the same CF projects differently [41]. More precisely, and following [41], we assume that experienced bakers can better evaluate the information signals than inexperienced ones. This takes the experienced bakers to adopt a more sophisticated decision-making process–that [41] define “ecological form of rationality”–that incorporates a mix of rationality and emotions. Acquiring experience should trigger the bakers to better investment decisions, leading to more resources collected by the winning projects.

BP4: With experienced backers, winning projects collect more funding.

## Experimental design and practice

In this section, we illustrate our experimental design: we first describe the contribution mechanism embodied in our setting, then present our four treatments, and finally briefly describe the participant samples and the experimental procedures.

### The contribution mechanism

The participants were asked to interpret the role of potential investors in projects launched on a CF platform. Specifically, they had to choose how to allocate a personal endowment of 150 experimental monetary units (EMU; where 50 EMU = 1€) among multiple CF projects seeking funding. As in real CF, a project was able to generate a return for its investors only if it collected enough funding to reach a given threshold (that is, we used the so-called provision point mechanism to simulate a real CF website, as in [46]). This setting captured the idea that investors choose not only whether to invest in a project or not, but also in which project to invest.

As anticipated, we investigated the role played by different degrees of competition introducing three between-subjects experimental conditions (Studies) that mimic the three funding mechanisms just described in the theory section. In Study 1, the participants could only choose which project to invest in but not the amount invested (i.e., they could either invest all their endowment at once in one of the available projects or not invest at all). We imposed high competition among projects by setting the funding threshold in a way that the participants could together afford to fund only one of the available projects. Specifically, we had 24 participants per session, each of whom was endowed with 150 EMU. Thus, there was a total of 3,600 EMU available altogether in each session, and we set the funding threshold at 1,950 EMU, so that only one project could reach the funding threshold. In Study 2, we kept the high competition among projects (meaning that only one project can be successful), but we allowed the participants to contribute to more than one project (i.e., they could divide their endowment and use only a part of it to invest in any of the given projects). Finally, Study 3 differed from Study 2 in that we imposed a lower level of competition among projects, by setting the threshold in a way that the participants could together afford to fund more than one project. The threshold was set to 1,350 EMU for each project. Recalling that in each session there was a total of 3600 EMU, this implies that more than one project (i.e., two) could reach the threshold. In all the studies, the participants were guided by the goal of identifying the winning project, because it was only through investing in a project that (eventually) reached the threshold that they could get a return on their investment. We call the process of selecting and funding a project a market session (MS). In each Study the participants played three consecutive MSs, with different sets of competing projects. At the beginning of each MS, the participants received their personal endowments and were informed that they could use them during any round of the current MS.

In each MS, the participants played for 10 periods (rounds)—being informed in each period after the first one of how much financing each project had collected so far. In each round, they decided whether and to which project to invest, while being aware that in line with actual CF mechanisms, contributions could not be withdrawn. Moreover, the participants could invest in a project even if it had reached the funding threshold.

The returns generated by the winning project were proportional to the amount a participant invested in that project. The participants who invested in a project that did not reach the threshold received a partial refund equal to 33% of the amount invested. This feature is aimed at mimicking the opportunity cost that investors incur in real non-financed CF projects, because of money locked-in until the end of the CF campaign [2].

### Projects, treatments and market sessions

Our experiment involved four treatments that differed in two dimensions, depending on whether we provided information about the horizontal attributes of the projects or not, and on whether the project returns were certain or risky (see Table 1). This 2x2 design allowed us to study the effect that information about horizontal attributes and risk might have on project selection and on the amount of funding a project can collect.

**Table 1:** Overview of the four treatments in the main experiment.

		*Returns*	
		Certain	Risky
*Horizontal Attribute Information*	**No**	Baseline	Risk
	**Yes**	Info	Combined

To create the projects (choose the characteristics for each project) and avoid arbitrary decisions regarding the choice of the parameters of information about horizontal attributes, risk, and returns, before our main experiment and using a different group of participants, we ran a separate project design experiment (PDE, for more details see S2 Appendix). The projects designed in the PDE experiment were used in the main experiment. Specifically, a project in our experiment is characterised by the following elements: *Name*—a colour randomly assigned to the projects to identify them; *Share of returns*—the (expected) value of the projects’ returns for each investor; *Lottery*—the level of risk characterising each project; *Horizontal attributes information*—information about some attributes of the project (the sponsor—an institution sponsoring the project—and the reputation) and some characteristics of the project’s designer (gender, education, and experience–in terms of number of experiments the designer previously attended). The characteristics of each project are reported in S2 Appendix.

All the treatments are repeated for each of the three studies, Study 1, Study 2, and Study 3.

### Treatments and market sessions

**Fig 1:**
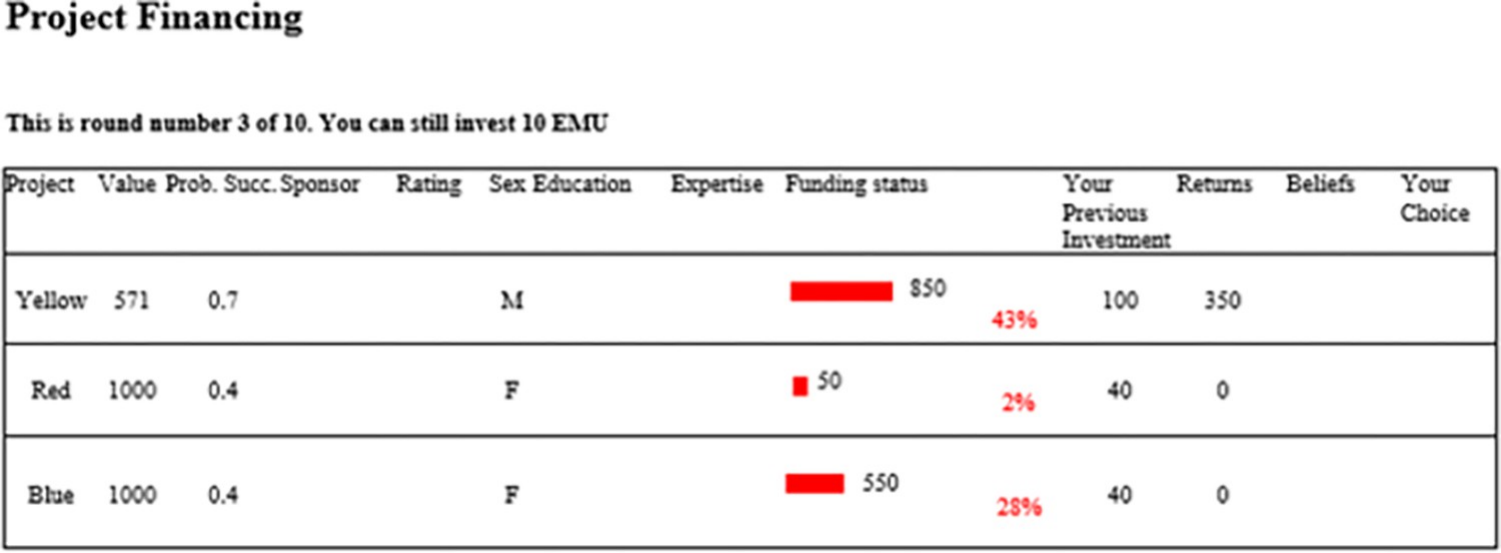
Choice window in the combined treatment. *Notes*: The participants could view only the values of one element of the horizontal attributes information at a time by selecting the corresponding name. In the example, ‘sex’ has been selected, showing the corresponding values for each project.

Our benchmark is the baseline treatment: projects were identified only by their names, and they had the same certain returns in case they reached the threshold. To analyse the impact of information about horizontal attributes and risk over project returns on the participants’ choices, we implemented three treatments. The *info treatment* differs from the baseline only in that we incorporated into the description of the projects the information about the horizontal attributes obtained from the PDE. The *risk treatment* differs from the baseline only in that we introduced risk over project returns, by linking each project to the corresponding lottery, which was run if the project was a winning one. We modelled the exogenous uncertainty as risk by informing the participants about the probability that a project would produce positive returns once it arrived in the market. The decision to experimentally model uncertainty as risk has been done to avoid introducing an uncontrolled factor into the experimental design. If the lottery succeeded, the investors gained the corresponding returns, otherwise, they got zero. Each lottery could have a different probability of generating a return (where lower probabilities corresponded to larger returns), but they had the same expected value, which was equal to the project returns in the baseline [47]. The *combined treatment* differs from the baseline in that we introduced both information about the horizontal attributes and risk over project returns. An example of choice window is shown in Fig 1 (see also S4 Appendix).

We ran each of the four treatments under the different funding mechanisms considered in the three studies. Therefore, by combining different decision mechanisms (the studies) with the effects of information about horizontal attributes and risk (the treatments), our experimental design enabled us to artificially reproduce a complete set of real CF contexts. In all treatments the participants were requested to play three market sessions (MS1, MS2, and MS3). In the first two market sessions the participants could choose among three projects identified by colours, while in the third market session the projects were six. We decided to double the number of the projects in the third market session to check if a greater range of options available would reduce the easiness and the speed of coordination. As anticipated in the theoretical section the aim for the introduction of more market sessions was to check if expertise impact on the participants’ behaviors, resulting in a better coordination and in higher amounts of money collected by the projects.

#### Participants and procedures

The PDE and the main experiment participants were randomly selected from a pool of Italian undergraduates at the University of Trento. The experiment was run at CEEL (Cognitive and Experimental Economic Laboratory) of the University of Trento (Italy). All experimental procedures followed the Declaration of Helsinki, and the General Data Protection Regulation (GDPR) of the European Union. Under these conditions, we do not need explicit approval from the Ethical Committee of the University of Trento (where the experiments were run). Participants were students at the same university recruited among the members of the laboratory database. The laboratory database contains only the entries of those subjects who indicated their consent. Those who signed the appropriate informed consent paperwork voluntarily participate in experimental research in the domain of social sciences. Thus, this experiment does not involve people unable to give informed consent, vulnerable individuals, or minors. Participants knew that the research was in the domain of social sciences and did not have any medical purpose or content. At the same time, they were aware that the data collected would be treated anonymously and analyzed on aggregate level without the individual data being traced back to the originator. In addition, we informed participants that sensitive personal data or genetic information would not be collected. Participants were paid a participation fee (show-up fee) and a fee proportional to their effort and results in the experiment, which was made clear before the start of the experimental session.

We recruited 19 participants for the PDE, and 288 participants for the main experiment, that is, 24 for each treatment in each study. Both experiments were programmed and conducted using oTree software [48]. After arriving at the laboratory, the participants were randomly assigned to a computer, and the instructions were read aloud by the experimenters (see S5 Appendix for the detailed instructions). Before starting the experiments, the participants had to answer a series of control questions to ensure a proper understanding of the instructions. In the main experiment, the participants were informed by the software when a new MS was starting. In the treatments with risk, feedback about the returns from the winning projects (i.e., the result of the lottery that took place if a project had reached the funding threshold) was given to participants only at the end of the experiment. Our payment structure was meant to mimic a typical reward from a CF campaign, in which the return would vary according to the amount invested in the winning project. The investment amounts were divided into ranges of less than 50 EMU, 50–99 EMU, 100–149 EMU, and a full 150 EMU. The returns were zero, one-third of the project return, two-thirds of the project return, and full project return, respectively. Clearly, in Study 1, participants could only get either zero or the full project return.

After the last MS, we used the bomb risk elicitation task (BRET; [49]) to elicit participants’ risk preferences. Here, the participants were shown 100 boxes. They were informed that 99 boxes contained 2 EMU each, while the remaining one contained a bomb that would explode and nullify the earnings for this part of the experiment. Each participant was then asked to collect as many boxes as they liked. The boxes were then opened. If the box with the bomb had not been selected, the participant’s earnings depended on the number of collected boxes; but if the bomb was among the boxes, the earnings were equal to zero. After the BRET, one of the three MSs was randomly drawn by the software, and the participants’ payoff (for determining the reward in Euros) was set equal to the return they received in the extracted MS. The total payment each participant received also included the EMU they collected from the BRET and the participation fee. The average payment was €11.50, including a €3 participation fee, for an experiment that took approximately one hour. After the experiment, the participants were asked to answer a brief questionnaire. Participants’ average age was 23 years; most of them were students of economics (61%), law (12%) and engineering (11%); and 51% was female.

## Results

This section presents the results from our experimental data. Given that in ‘*the context of crowdfunding platforms*, *funding success is a multifaceted concept*’ ([8], p. 961), we considered whether and how a project reached the funding threshold and the time (number of rounds) needed to reach the threshold. Our experimental design allows us to analyse two further aspects of a CF platform: the institutional framework, here depicted by the three studies, and the crowd expertise, which is induced on the participants through the three MSs.

### Winning projects

We start the analysis by examining the winning projects in the different MSs. Specifically, we assess whether introducing information about the horizontal attributes and/or risk impacts project selection. Tab. 2 reports the amount of funding collected by each project in the different treatments, together with a summary of the characteristics of each project.

According to the literature, if information about horizontal attributes is effective in making a project salient, a project with the same characteristics should be chosen across the different MSs. Tab. 2 shows that, in the *info* treatment, the same projects were indeed selected in all the MSs of both Studies 1 and 2. Moreover, winning projects in the different MSs always shared the same attributes: they were sponsored by a bank and were designed by an experienced, male, economics student (in the following, we call this project a ‘focal’ project). This result is in line with [10], who find that having a background in economics and management, and being experienced are effective signals of project quality, thus increasing the likelihood of success. The same does not hold in Study 3, even if the focal project was the non-winning project with the highest funding in all MSs.

The literature also highlights that those projects with a lower level of risk should be more likely to be selected [50, 51]. Table 2 reports the probability of each project generating returns (the column ‘risk’), and shows that, accordingly with the literature and regardless of the degree of competition, in the *risk* treatment participants chose low-risk projects in both MS1 and MS2. Despite this literature-coherent result, in MS3 they switch their behaviors, choosing higher risk ones. However, on average, low-risk projects were able to collect more funding than the high-risk ones (1,940 vs. 333 EMU).

**Table 2:** Total funding for each project.

Market session (MS)	Project	Risk	Horizontal Information								Amount Collected								
			Projects		Designer			Baseline			Info			Risk			Combined		
			Sponsor	Rating	Exper.	Educ.	Sex	S1	S2	S3	S1	S2	S3	S1	S2	S3	S1	S2	S3
MS1	Yellow	0.7	Bank	4	15	Econ.	M	300	412	2767	3450	3052	262	3300	2368	1610	3450	2768	1995
	Red	0.4	Insur.	4	8	Soc.	F	150	3006	432	150	265	107	150	601	1724	150	604	266
	Blue	0.4	Univ.	2	5	Law	F	3150	182	299	0	209	3187	150	427	216	0	147	1184
MS2	Yellow	0.5	Univ.	9	6	Law	F	3450	15	3249	150	65	3555	2850	416	607	750	3068	25
	Red	0.4	Bank	2	20	Econ.	M	0	3570	58	3450	3432	40	300	169	48	2700	60	457
	Blue	0.6	Insur.	5	2	Eng.	M	0	15	203	0	4	5	300	2980	2795	0	259	3018
MS3	Yellow	0.5	Univ.	9	6	Law	F	0	0	1845	0	171	0	0	44	0	0	29	1
	Red	0.5	Curia	6	8	Law	F	0	394	1587	150	21	3430	2550	207	2481	150	245	153
	Blue	0.6	Univ.	3	3	Eng.	M	0	3106	2	0	1	0	150	372	0	0	48	710
	Green	0.4	Bank	2	20	Econ.	M	0	0	57	3450	3355	170	0	15	38	2100	46	2421
	Purple	0.6	Insur.	5	2	Eng.	M	3300	0	2	0	0	0	0	30	18	150	60	1
	Orange	0.7	District	9	5	Econ.	M	150	0	2	0	2	0	300	2732	1063	300	3120	71

The participant choices in the *combined* treatment allowed us to disentangle the relative impact of the information about the horizontal attributes and the level of risk in the selection of the winning project. We observe (Tab. 2) that, regardless of the study and level of risk, the focal projects were mostly preferred (two-thirds of the time across MSs). This suggests that information about horizontal attributes has a dominant role over risk in determining which project will win.

**Result 1**: *In all the studies*, *when information about the horizontal attributes was made available*, *the participants were more likely to choose a focal project (i*.*e*., *a project sponsored by a bank and designed by an experienced male economics student)*, *independently of the project’s risk level*. *However*, *when information about horizontal attributes was unavailable and project returns were risky*, *the participants were more likely to choose the lowest-risk projects*.

It follows that the results support *STP1b*, *BP1a*, *BP1b* and *STP2b*.

### How much funding was collected?

In this section, we provide a detailed analysis of the amount collected by the winning project.

### Overall contributions across treatments

**Fig 2:**
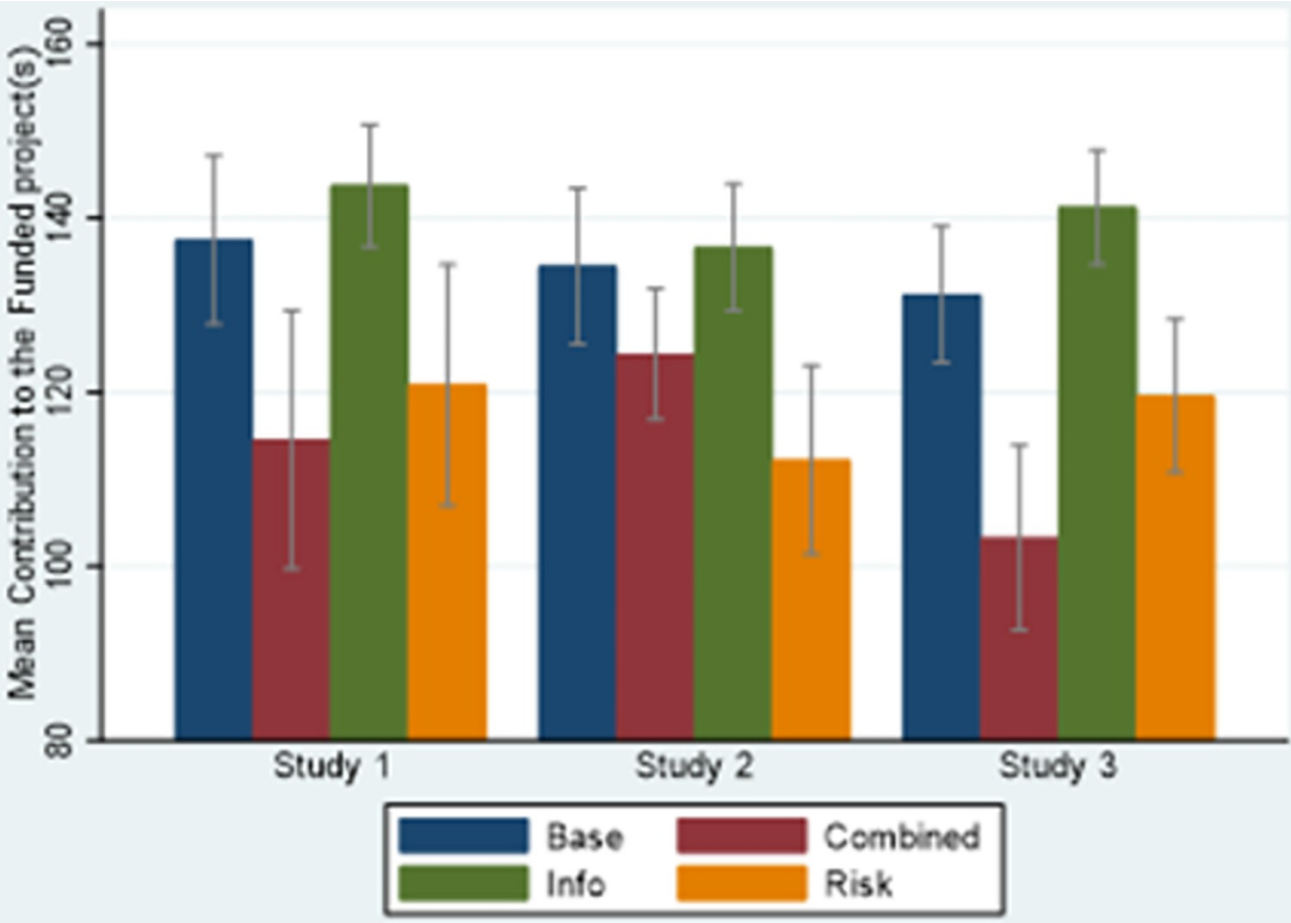
Average contributions to the funded project(s) in the four treatments over the three studies (with standard error mean bars).

Fig 2 provides a visual analysis of the average amount invested in the winning projects of each treatment in each study.

Looking at Fig 2, the highest average contributions are collected in the *info* treatment, while both the *risk* and *combined* treatments collected the lowest contributions. To check the significance of these results, reference can be made to Table 3, reporting the results from three random effects generalised least squares models (one model for each study), where the dependent variable is the individual contribution to the winning project(s) and the independent variables are the treatments, with the *baseline* treatment as our baseline category. We introduce random effects to check for repeated decisions.

**Table 3:** Random effects generalised least squares regression on contributions by study.

	Study (1)	Study (2)	Study (3)
Variables	Model 1	Model 2	Model 3
Combined	-22.92*	-10.08	-27.97***
	(9.507)	(8.245)	(6.871)
	(0.016)	(0.221)	(0.000)
Info	6.250	2.181	10.06
	(9.507)	(8.245)	(6.871)
	(0.511)	(0.791)	(0.143)
Risk	-16.67+	-22.25**	-11.64+
	(9.507)	(8.245)	(6.871)
	(0.080)	(0.007)	(0.090)
_cons	137.5***	134.5***	131.2***
	(6.723)	(5.830)	(4.859)
	(0.000)	(0.000)	(0.000)
*N*	288	288	288

*Notes*: The dependent variable is the individual total contribution to the winning project. Specifically, the dependent variable in Studies 1 and 2 is the individual contribution to one winning project because only one of the available projects can gather enough support to reach the funding threshold. The dependent variable in Study 3 is the contribution to one or two winning projects. In fact, in Study 3, up to two projects can reach the funding threshold. Our baseline category is the baseline treatment. N = 288; number of independent observations = 96. Standard errors in parentheses (in the second line for each variable); p values in parentheses (in the third line for each variable)

+ p < 0.1 + * p < 0.05

** p < 0.01

*** p < 0.001.

As shown in Table 3, compared with the baseline treatment, introducing information about horizontal attributes does not significantly increase the amount invested in the winning project, while introducing risk leads to significantly lower contributions. This is in line with the ‘certainty effect’ [52], indicating a tendency to give more weight to certain outcomes than to risky ones. In our setting, this translates into higher contributions in the *baseline* (with deterministic outcomes) than in the *risk* treatment. This finding is also in line with the results presented by [18], who examine how backers responded to a new policy launched by Kickstarter in 2012, requiring the project creators to disclose the possible risks involved in their projects; they find that risk disclosure decreased contributions. Introducing a combination of information about risk and horizontal attributes significantly reduces contributions in Studies 1 and 3. The results are robust. A series of Mann–Whitney tests (see S3 Appendix) confirm these results, with the only two exceptions being the difference between *baseline* and *info* in Study 3 that had significance at p = 0.0369 and the difference between *baseline* and *combined* in Study 2 that had significance at p = 0.0001 level.We summarise these findings as follows:

**Result 2**: *Providing information about horizontal attributes does not significantly increase contributions*, *while risk significantly decreased contributions*. *Risk*, *along with information about horizontal attributes*, *significantly decreased contributions in Studies 1 and 3*.

Overall, result 2 supports *BP2* and seems to offer a broad support to predictions *STP1a* and *STP2b*. Of course, *STP1a* is not confirmed if strictly applied.

#### Contributions over the three market sessions

Here, we investigate whether and how the effect of information about horizontal attributes and risk varies because of the expertise acquired by the participants across MSs. We will define participants as ‘inexperienced’ when they participate in the first market session (MS1) and as ‘experienced’ in the consecutive market sessions (MS2 and MS3).

Table 4 reports the results of two random effects generalised least squares models for each study. Models 4, 6 and 8 control for the treatments and the MSs. Comparing the results in Tab.3 and in Tab.4, we notice that adding a control variable for the MS does not change the significance or sign of the treatment coefficients, thus confirming the above-discussed role of the treatments on the amount invested. It is also worth noting that compared with MS1, investments in the winning project were higher in MS2 of Studies 2 and 3 and in MS3 of Study 2, while they were lower in MS3 of Study 1. These results seem to confirm what is reported by the literature about the importance of the role played by the backers’ degree of expertise, suggesting that different MSs with experience effects have an impact on the choices of the participants.

**Table 4:** Random effects generalised least squares regression on contributions over the three MSs.

	Study 1		Study 2		Study 3	
Variables	Model 4	Model 5	Model 6	Model 7	Model 8	Model 9
Combined	-22.92*	14.05	-10.08	-8.570	-27.97***	-31.53**
	(9.507)	(14.264)	(8.245)	(10.671)	(6.871)	(10.181)
	(0.016)	(0.325)	(0.221)	(0.422)	(0.000)	(0.002)
Info	6.250	11.82	2.181	6.549	10.06	16.70+
	(9.507)	(14.295)	(8.245)	(10.839)	(6.871)	(10.138)
	(0.511)	(0.408)	(0.791)	(0.546)	(0.143)	(0.099)
Risk	-16.67+	7.564	-22.25**	-23.77*	-11.64+	23.23*
	(9.507)	(14.262)	(8.245)	(10.731)	(6.871)	(10.092)
	(0.080)	(0.596)	(0.007)	(0.027)	(0.090)	(0.021)
MS2	-9.375	12.50	19.33***	23.50**	13.90**	20.08*
	(6.747)	(13.173)	(4.264)	(8.571)	(4.965)	(9.131)
	(0.165)	(0.343)	(0.000)	(0.006)	(0.005)	(0.028)
MS3	-20.31**	6.250	11.66**	4.167	5.010	27.71**
	(6.747)	(13.173)	(4.264)	(8.571)	(4.965)	(9.131)
	(0.003)	(0.635)	(0.006)	(0.627)	(0.313)	(0.002)
Combined#MS2		-43.75*		-11.00		22.54+
		(18.629)		(12.122)		(12.913)
		(0.019)		(0.364)		(0.081)
Info#MS2		-12.50		-7.667		-4.750
		(18.629)		(12.122)		(12.913)
		(0.502)		(0.527)		(0.713)
Risk#MS2		-31.25+		2.000		-42.54***
		(18.629)		(12.122)		(12.913)
		(0.093)		(0.869)		(0.001)
Combined#MS3		-62.50***		10.50		-9.958
		(18.629)		(12.122)		(12.913)
		(0.001)		(0.386)		(0.441)
Info#MS3		-6.250		8.458		-17.58
		(18.629)		(12.122)		(12.913)
		(0.737)		(0.485)		(0.173)
Risk#MS3		-37.50*		11.00		-63.25***
		(18.629)		(12.122)		(12.913)
		(0.044)		(0.364)		(0.000)
BRET		-0.381*		-0.133		0.149
		(0.193)		(0.177)		(0.127)
		(0.049)		(0.452)		(0.240)
Female		-10.62		-14.90*		-6.928
		(6.878)		(5.852)		(5.073)
		(0.122)		(0.011)		(0.172)
Constant	147.4***	152.3***	124.1***	137.4***	124.9***	113.6***
	(7.770)	(13.718)	(6.329)	(11.278)	(5.641)	(9.211)
	(0.000)	(0.000)	(0.000)	(0.000)	(0.000)	(0.000)
*N*	288	288	288	288	288	288

*Notes*: The dependent variable is the individual total contribution to the winning project. Our baseline categories are the baseline treatment, the first market session and male. N = 288; number of independent observations = 96. Standard errors in parentheses (in the second line for each variable); p values in parentheses (in the third line for each variable)

+ p < 0.1 + * p < 0.05

** p < 0.01

*** p < 0.001.

To investigate whether and how the treatment effects depend on the degree of expertise of the participants, in Models 5, 7 and 9, we add interaction terms between the treatments and MSs. We also control for gender (because of previous evidence of higher female risk aversion, e.g., [53]), and for the impact induced on funding decisions by risk attitudes (variable BRET). Tab. 4 shows that both gender and BRET do not significantly affect contributions, with two exceptions: more risk-loving participants (higher values of the variable BRET) invested less in Study 1, while female participants invested less in Study 2.

**Fig 3:**
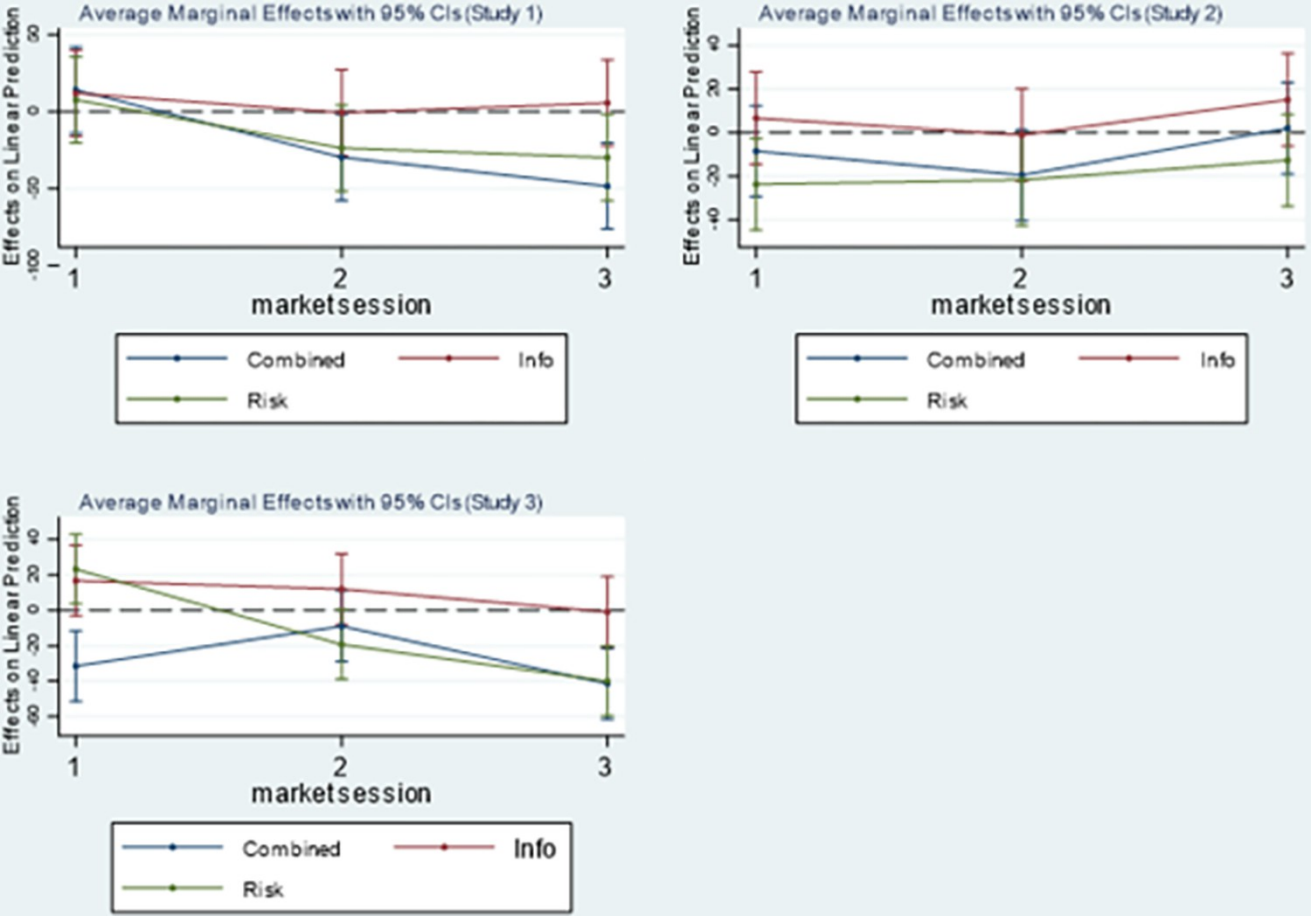
Marginal effect of the treatments (*info*, *risk*, *combined*) on the amount invested relative to the baseline in each of the three market sessions. *Notes*: Plots of the marginal effect from Models 5, 7 and 9 estimated setting the remaining variables as they were observed.

To better understand the results of Models 5, 7 and 9, we rely on marginal effect analysis [54, 55]. Fig 3 graphically shows the marginal effect of the treatments on the amount invested relative to the baseline for the three MSs and for each study.

We first note that, although the marginal effect of the *info* treatment is always positive, it is significant only in MS1 of Study 3 (p = 0.099). Moving to the *risk* and the *combined* treatments, in Study 1 both marginal effects are not significant in MS1, but they are negative and significant in MS2 (p = 0.097 and p = 0.037, respectively) and in MS3 (p = 0.036 and p = 0.001, respectively). In Study 2, the marginal effect of the *combined* treatment is not significant in MS1, while the marginal effect of the *risk* treatment is negative and significant (p = 0.027). In MS2, the marginal effect of the *risk* and *combined* treatments are negative and significant (p = 0.043 and 0.067, respectively), while they are both not significant in MS3. Concerning Study 3, in MS1, the marginal effect of the *combined* treatment is negative and significant (p = 0.002), while the marginal effect of the *risk* treatment is positive and significant (p = 0.021). In MS2, the marginal effect of the *risk* treatment is negative and significant (p = 0.056), while the marginal effect of the *combined* treatment is not significant. In MS3, the marginal effect of both the *combined* and *risk* treatments are negative and significant (p = 0.000). Results are aligned with the Mann–Whitney tests (see S3 Appendix).

**Result 3**. *Information about horizontal attributes increased contributions in Study 3 only when the participants were inexperienced*. *Risk decreased contributions in all the studies when the participants were experienced*. *Risk had a significant effect on the inexperienced participants in Studies 2 and 3*, *where the effect was negative in Study 2 and positive in Study 3*. *The combination of risk and information about horizontal attributes always decreased contributions*, *especially when the participants were experienced*.

These results clearly support BP4 but in a composite way that does not allow to reach strong conclusions. More specifically, it is not possible to give support to some literature evidence that suggest that experienced bakers should be more efficient in evaluating and using the available information signals [56].

#### Horizontal attributes and risk

Our experimental design allows us to compare the role of information with certain and risky returns and the role of risk in the presence or absence of information about the horizontal attributes. S3 Appendix reports the results from a series of Mann–Whitney tests in which we compare the average amount invested in the *combined* treatment with the average amount invested in the *risk* and *info* treatments. Regardless of the study, providing a combination of risk and information about horizontal attributes (*combined* treatment) leads to significantly lower contributions compared with the *info* treatment. Moreover, Mann–Whitney tests do not indicate a significant difference between contributions in the *combined* and in the *risk* treatment, with the only exception of MS1 in Study 3, where contributions in the *combined* treatment are significantly lower than those in the *risk* treatment. Taken together, these results show a dominant role of risk over horizontal attributes in determining the amount invested in the winning project. This consideration, together with the analysis provided in the theoretical Section, allows us to summarise our fourth result:

**Result 4**: When returns are risky and information about the horizontal attributes is provided, information about horizontal attributes prevails in determining which project wins. However, risk prevails in determining the amount invested in the winning project.

#### The speed of coordination

Previous research has shown that projects collecting more funding from the very beginning of a campaign are more likely to reach the funding threshold [57–59]. Thus, the amount of collected funding is not the only indicator of the likelihood of the success of a project, as the speed of funding also plays an important role. For this reason, we now analyse whether our treatments influence the time it takes for a project to reach the threshold (i.e., the number of rounds a project needs to be successfully funded).

**Fig 4:**
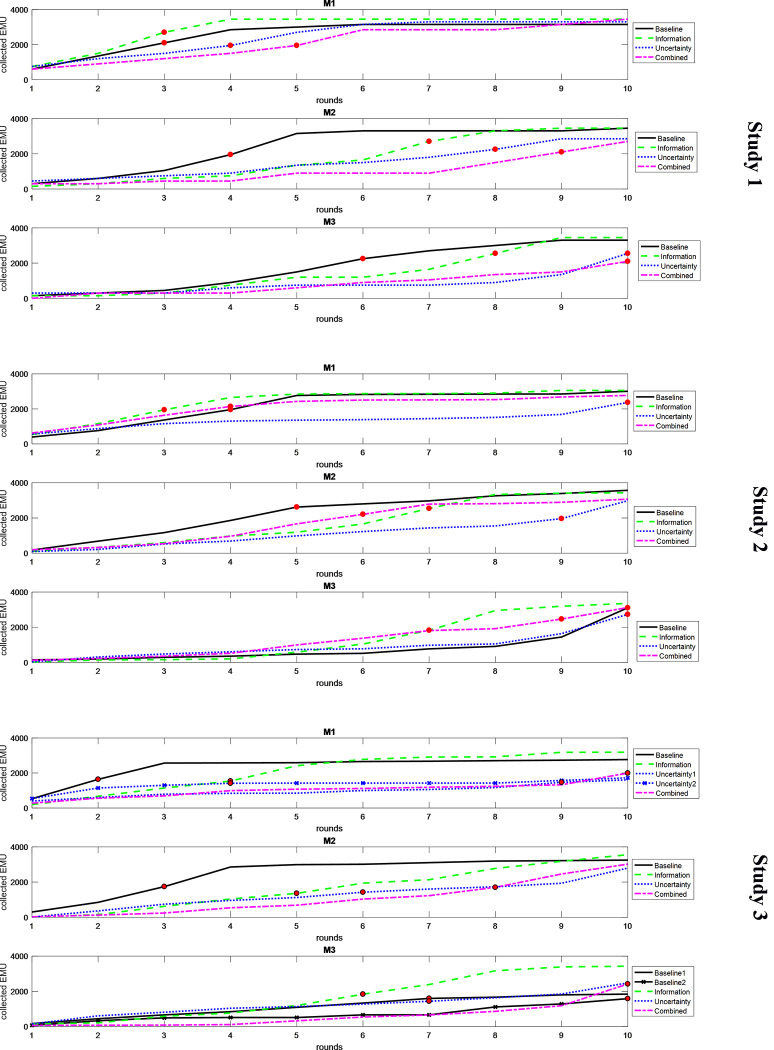
Cumulated funding of each winning project. *Notes*: Cumulative funding collected by each winning project across treatments in each round of the three market sessions (M1, M2 and M3). Dots indicate when the funding threshold was reached.

Fig 4 shows the amount of cumulative funding for the winning projects across treatments over the three MSs of each study.

Regardless of the study and treatment, it took more rounds for a project to reach the funding threshold passing from MS1 to MS2 and MS3. The presence of information about horizontal attributes helped a project reach the funding threshold faster only in MS1 of Studies 1 and 2, and in MS3 of Study 3. The introduction of risk slowed coordination around the winning project from the first MS. This effect was particularly sharp in Studies 2 and 3. Thus, besides decreasing the amount that a project can collect, the presence of risk also slows down the funding process.

**Result 5**: *Experienced participants tend to wait more before investing*, *regardless of the study and treatment*. *Introducing risk increased the number of rounds needed for the winning project to reach the funding threshold*, *regardless of the experience participants have*.

## Discussion and conclusions

We investigated the determinants of project success on CF platforms at the level of an individual decision maker. Our study contributes to the growing literature on experimental decision-making by investigating the influence of signals from entrepreneurs seeking funding on investors’ decisions. We extend recent field experiments [13, 23] that investigate the effects of signals on reducing information asymmetry for investors, and we explicitly account for exogenous risk to examine how it interacts with horizontal attributes to affect project success.

In contrast to previous laboratory experiments, mainly focused on donation-based CF modelled as a public goods game [2, 24–27], our study employs a dynamic coordination game with a threshold to model CF, capturing the dynamics of various CF types, such as Equity-based CF.

Our study also provides insights into the interplay between horizontal attributes and exogenous risk in a competitive CF environment where multiple projects compete for limited funding. Previous research has primarily focused on fundraising within individual campaigns, with little attention paid to cross-campaign dynamics [29]. Our unique experimental design enables us to investigate this phenomenon and observe the causal effect of horizontal attributes and risk on investor decision-making, taking into account different levels of competition among projects.

Even though we designed our experiment with the explicit intent of simulating a CF platform, we want to highlight that this is not the only possible interpretation, and our setting can be used to study other phenomena. An example is a market for network goods, that is, goods whose utility increases as more people buy the same good, or standard adoption, in which a standard has a higher utility the more people use it. In such cases coordination is an issue [60], as there can be competition between different technologies, and a consumer might buy not the technology he deems the best, but the one he thinks that other people will acquire the most. In this sense, our projects can be considered as goods that generate a positive utility only if a critical mass of other consumers would choose the same project. As such, our results should not be confined to the sole environment of CF.

Our research offers valuable insights into the complex nature of CF and its underlying mechanisms. Our results shed light on several key debates in the entrepreneurial financial literature and on the important question of what mechanisms drive individual decision makers to fund a project.

First, we find that information about the horizontal attributes of the project and its designer are crucial in driving financing choices towards a common target project because participants with such information chose the same project across MSs more often. However, although information about horizontal attributes has a focalising role favouring participant coordination, it slows down the process of coordination. A purely speculative explanation of this phenomenon is that providing the participants with this kind of information generated an informative white-noise effect, increasing their cognitive load and distracting them from coordinating quickly, instead making them devote themselves to identifying the ‘focal’ attributes of the projects. Increasing the cognitive task carried out by the decision makers translates into a mechanism of slowing down the coordination process.

Second, the presence of risk (*risk treatment*) promotes choosing lower-risk projects, across MSs and studies, in line with previous results [10, 50, 51]. Importantly, however, we must remember that this result was always true in MS1 and MS2 but that a turn-around was observed in MS3 with a certain prevalence of choices favouring the riskier projects. The rationale behind these findings can be linked to the existence of two distinct cognitive mechanisms at work during MS3: the first linked to a lottery ‘portfolio’ effect and the second to self-confidence acquisition. Regarding the first mechanism, it could be thought that the participants ‘tied’ the lotteries connected with the projects to one another through the MSs. Hence, they could build a portfolio of projects (i.e., lotteries connected with the projects) that best suited their preferences. Moreover, we found that *risk* reduced the amount invested in the projects, which can be considered a result of the cognitive cost that the introduction of risk entails in any decision-making process. This result aligns with the ‘certainty effect’ [52], confirming previous empirical evidence on the negative effect of the risk-disclosure policy adopted by Kickstarter on funding contributions [18]. This finding has practical implications for CF platforms, suggesting they should be very careful in designing their risk-disclosure policies, as providing information about risk may have a negative effect on funding decisions. Together with adding a requirement for information about possible risks, the CF platform providers can require project creators to identify ways of mitigating the identified risks—this idea has been voiced before. If information about risk mitigation is disclosed, investors could perceive the outcome of their investment to be more certain, thus increasing their contributions because of the aforementioned ‘certainty’ effect.

Third, we examined the role of information about horizontal attributes in the presence of risky returns (*combined treatment*), finding that information about horizontal attributes prevails over risk in the *project selection process*, as participants were more likely to choose a focal project, regardless of the project’s risk level. However, risk prevailed over information about horizontal attributes in the *investment decision process*, so that when information was provided, contributions in the presence of risk were lower than those in the absence of risk. Contributions were not significantly different in the *risk* and *combined* treatments, enforcing that risk has a dominant role in determining the amount invested in the winning project. The main takeaway here is that when information about both project’s risk and horizontal attributes is disclosed, particular attention should be paid to risk disclosure (and risk mitigation).

Finally, experience changes investment decisions in that experienced participants make their choices later than inexperienced ones; this points to the possibility that what we call *rational decision-making* may be something that needs to be learned. This is an interesting avenue for further research that was not explored within the present research.

Our results are robust across the three studies, with the only exception being MS1 of Study 3, where both information about horizontal attributes and risk significantly increased contributions to the winning project(s). This suggests that when the equilibrium selection problem is more severe, the presence of a signal that can make a project focal (in terms of human capital or risk) is more valuable, especially when the participants are inexperienced. As such, our results contribute to a better understanding of the micro-foundations of what determines investment decisions in CF.

By explicitly accounting for exogenous risk and examining its interplay with horizontal attributes, our research provides valuable insights into how project creators can design their campaigns to attract more funding. Indeed, we provide a better comprehending of which information influences investment decisions and what factors influence the amount and speed of collected funding. These insights can inform the designers of CF platforms and campaigns, potentially increasing their effectiveness and helping entrepreneurs and creators raise the necessary capital for their projects.

Additionally, our experiment simulates a quasi-realistic CF platform, allowing for a better understanding of how investors behave when presented with multiple investment options. By incorporating our findings into their investor and client interfaces, platforms can provide investors with relevant information that will help them make better investment decisions.

Ultimately, our findings can enhance the effectiveness of crowdfunding as a valuable investment tool by providing investors with important insights into the factors that influence crowdfunding success rates. These include attributes related to the project, the project’s creator, and the risk level of project returns. Armed with this knowledge, investors can make more informed decisions about which projects to fund, leading to greater overall success rates and increased confidence in the crowdfunding platform.

## Supporting information

S1 Appendix Theoretical predictions and resources allocation. (PDF)

S2 Appendix The project design experiment. (PDF)

S3 Appendix Results of Mann–Whitney tests. (PDF)

S4 Appendix Examples of choice windows. (PDF)

S5 Appendix Instructions. (PDF)
