# Supplementary material for: Ignorance is bliss? Information and risk on crowdfunding platforms
Source: PLoS One. 2023 Jun 16;18(6):e0286876. doi: 10.1371/journal.pone.0286876 (PMC10275436; doi:10.1371/journal.pone.0286876)
Supplement: S1 Appendix — (PDF) [file pone.0286876.s001.pdf]

S1 Appendix A. Theoretical predictions and resources allocation

A.1 Theoretical Framework

We consider a game played among $N \geq 2$ potential investors of $J \geq 2$ competing identical projects. Each player has an initial endowment equal to e, and each project has a funding threshold, k, so that it will generate a return for its investors only if it collects enough funding to reach the threshold – in this case, we call it a "winning project". The investment of player i in project j is $x_{ij} = b_{ij} e$, with $0 \leq b_{ij} \leq 1$ and $\sum b_{ij} = 1$. Then, for each project j, if $\sum x_{ij} \geq k$, so that j is a winning project, player i will get a return equal to $b_{ij} R$; otherwise, he will get a partial refund equal to $b_{ij} c$, with $c < e < R$. As there are N players, each of them with an initial endowment equal to e, there can be, at most, Ne/k winning projects. Thus, the lower Ne/k is, the higher the competition among projects.

We consider three different funding mechanisms (FMs): in FM1, there can be, at most, one winning project ($Ne/k < 2$), and players cannot divide their endowments into different investment decisions ($b_{ij} \in \{0,1\}$); FM2 differs from FM1 in that players can invest in several projects ($b_{ij} \in [0,1]$); FM3 differs from FM2 in that there can be more than one winning project ($2 \leq Ne/k < 3$).

A.1.1 Static case

Assume first that the game is played simultaneously. Let $x = (x_1, \ldots, x_N)$ be the profile of the players' investment decisions, where $x_i = (x_{i1}, \ldots, x_{iJ})$. The game has two types of pure equilibria:

I) *not investing*: $b_{ij} = 0$ for all players and all projects, and players' payoff is equal to e;

ii) *full investment*: all players invest their full endowment. If only one project can be funded, then all players will invest in the (same) winning project. If more than one project can be funded, there are equilibria in which players invest in different (winning) projects. In any case, players' payoff is R.

Following Heinemann et al. (2009), who considered the case of $J = 1$, one can show that there are two types of mixed equilibria:

i) *type m1*, in which all players mix among J' projects ($1 \leq J' \leq J$), with the same probability, equal to p, and they choose not to invest with probability $1 - J' p$. For example, if $J' = 2$, p solves:

$$e - c = (R - c)\left(1 - Cum\left(Mult(N-1, p, p, 1-2p), k-2, N-1, N-1\right)\right) = (R - c) q(p) \quad (2),$$

where Cum is the cumulative multinomial distribution and $q(p)$ is the probability that a project reaches the threshold (the probability that at least $k-1$ over $N-1$ players will invest in project j, from the point of view of a player who has not yet invested). Players' (expected) payoff is e;

ii) *type m2*, in which players mix among J' projects ($1 < J' \leq J$), with a probability equal to $1/J'$, and the (expected) payoff is $c + (R - c) q(1/J')$.

It is worth noticing that we are assuming risk neutrality or, alternatively, that we are assuming that the players' preferences towards risk are homothetic.

A.1.2. Dynamic case

Consider now the case in which players have T periods to finance a project: if, by the end of the game, a project collected enough funding to reach the threshold, it will generate a return for its investors; otherwise, its investors will get a partial refund, as in the static game. However, in the dynamic game, players have the option to wait and observe the amount collected by each project.

Let x_{ij}^{t} be the decision of player i in period t regarding project j. The funding allocation observed in period $t>1$ is $G(t)=\{G_j(t)\}$, where $G_j(t)=\sum\sum x_{ij}^{r}$. Then, a strategy $x_i=\{x_{ij}^{t}(G(t))\}$ for player i must specify a decision for every project *j*, every period *t*, and, if $t>1$, any allocation $G(t)$. As with the static case, there are two types of pure equilibria:

I) *not investing*: the equilibrium in which nobody invest survives in the dynamic setting, but only provided that the game is short enough. Specifically, it must be $T<k/e$. To see why, consider the simple case of 4 players and a threshold equal to $3e$. In the last period, if a player observes $G(T)=2e$, he will invest for sure. Knowing this, if player i observes $G(T)=e$ in period $T-1$, and he knows that at least another player can still invest, he will invest, as he knows that in the next period the other player will invest also. This implies that in $T-2$ a player will invest even when he observes $G(T)=0$, provided that at least 2 other players also have the resources to invest in the next period. Then, in this example, whenever $T\geq 3$, the not investing strategy is weakly dominated;

ii) *full investment:* this set of equilibria becomes larger as we increase the length of the game, as any strategy profile such that, by the end of the game, all players invest their full endowment in one (or more, if possible) winning project is an equilibrium, no matter the order in which decisions are taken.

The dynamic game has also multiple mixed equilibria. An example is the equilibrium in which nobody invests until the very last round, and then one of the mixed equilibria of the static game is played. Unfortunately, in order to give a full description of the mixed equilibria, one should consider a large number of possible combinations of previous histories and strategies, which makes the problem intractable, but it is still possible to have some insights on the structure of these equilibria:

iii) type m1: players mix between investing in (at least) one project, and not investing. There are multiple equilibria of this type: in any period in which no project reached the threshold yet, and in which there are still resources available to finance it, it is possible for players to mix in that period.

iv) type m2: players mix between two (or more) projects. In a dynamic setting, the only equilibrium of this type requires players not to invest until the very last round, and then play (one of) the mixed equilibria of type m2 of the static game. To see why, just notice that in any period before the last one, if a player knows that everybody will choose in the current period, it is optimal for him to wait for the next period, in order to observe which project – if any – is able to reach the threshold, and then make his decision, of course this still leaves a lot of equilibria.

A way to restrict the number of the equilibria is to consider strategies that are stationary and monotonic. This restriction implies that we are assuming that the players once have chosen a given strategy, should always stick on it and that any adjustment goes only into the direction of increasing the amount of money invested in those projects which have been already financed. Introducing this assumption we have: given $G_j(t)=G$, if a player invests in project j in period t, he would invest in project j whenever $G_j(t')\geq G$; if instead he chooses not to invest in period t, he would not invest whenever $G_j(t')\leq G$; finally, if a player mixes in period t, we require that he never invests with probability one, if $G_j(t')<G$, and he never invest with probability zero, if $G_j(t')>G$. Stationary strategies directly imply that also equilibria of *type m2* are ruled out. We are left with two types of equilibria: full investment, and mixed equilibria. The

important difference between the two types of equilibria is that, while in the full investment equilibrium the "project selection" problem is not solved, in the mixed equilibrium the problem is solved through the specific sequence of play. In fact, the first players have the chance to address on a specific project their investments, creating in this way an "attractor" on the project chosen. Of course, accordingly with our original assumption of absence of idiosyncratic preferences, this is equivalent to say that every project has the same chances to become the winning one. Indeed, one might speculate that, when players do not have any reason to prefer a project to the other, as they all give the same return, one way to solve the project selection problem is to play the mixed equilibrium. This implies that whether a project is able to reach the threshold would solely depend on what happens during the rounds of the game.

A.2 Resources Allocation

According to our theoretical predictions, if a project reaches the threshold in the treatments with certain returns, it is never optimal to keep the endowment, while if returns are risky, it might be optimal to make a partial investment or not invest at all. We now investigate whether this is the case, or if the lower level of contributions was due to the participants choosing an unsuccessfully funded (i.e., losing) project. Fig. A1 reports the portion of the initial endowment that the participants invested in winning projects and in other losing projects, or did not invest, for each treatment and in each study. On average, horizontal attribute information decreased the amount invested in losing projects (around 5%), while risk led to a greater waste of endowment (more than 15%). Thus, even if more participants decided not to invest at all when returns were risky, the lower amounts of funding collected by winning projects were due to a larger investment in projects that did not reach the threshold.

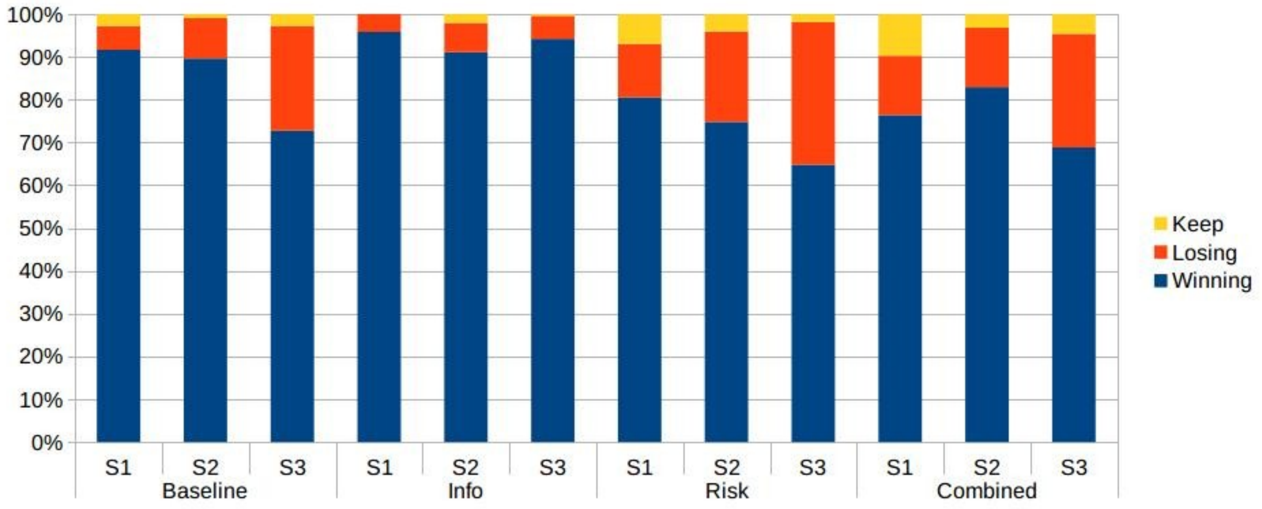

Figure A1. Resource Allocation by Treatment and by Study.

Notes: The figure shows the portion of endowment that, on average, participants decided to keep (i.e., not invested) and the portions invested in winning and losing projects (i.e., projects that did not reach the threshold) for each treatment and for the three studies (S1, S2, and S3). Aggregate data over the three market sessions were considered. N = 72.
