# Supplementary material for: Ignorance is bliss? Information and risk on crowdfunding platforms
Source: PLoS One. 2023 Jun 16;18(6):e0286876. doi: 10.1371/journal.pone.0286876 (PMC10275436; doi:10.1371/journal.pone.0286876)
Supplement: S2 Appendix — (PDF) [file pone.0286876.s002.pdf]

S2 Appendix B. The Project Design Experiment

In the Project Design Experiment participants played the role of project's designers (PDs). More precisely, they were asked to design, in their opinion, optimally interesting projects by choosing values for given characteristics, knowing that these projects would later on be used in our main experiment. Specifically, and in accordance with the literature examined and discussed in Section 2, a PD had to select a value for each of the following characteristics:

a) *Profit sharing*: the share of profits he is willing to give to the investor if the project is successful. The PD could choose a value from a minimum of 10% to a maximum of 60%, in steps of 10%.

b) *Sponsor*: an institution sponsoring the project. The PD had to choose one sponsor among several proposed institutions, i.e. “Bank”, “Local government”, “Insurance Company”, “the Church”, and “the University”.

c) *Risk-level*: a lottery associated with the project, which is a combination of the probability that the project will generate positive returns in case it reaches the threshold, with the amount of such returns. The PD had to choose one among a set of nine predetermined choices[1] , all with an expected value of 1000 experimental monetary units (EMU).

After all the participants had designed their projects, we ran a “beauty contest”[2]: each PD had to select up to three projects that, in their opinion, would be selected by the highest number of PDs as the one that most likely would reach the funding threshold in the main experiment. With the results of this vote, we obtained a ranking that we interpreted as the reputation of the project among the PDs; this is the second component of the horizontal attributes information. We also collected (to be then added to the description of the projects) horizontal attributes information regarding the PDs: gender, educational level (degree program), and previous experience (in terms of participation in previous experiments).

With the PDE’s results, we then performed a cluster analysis to build homogeneous groups of projects based on their attributes. From these clusters, we then selected the projects to be used in each MS of our main experiment (see Tab. B1).

1 The available choices were: (10%, 10000 EMU), (20%, 5000 EMU), (30%, 3333 EMU), (40%, 2500 EMU), (50%, 2000 EMU), (60%, 1667 EMU), (70%, 1429 EMU), (80%, 1250 EMU), (90%, 1111 EMU).

2 The term “beauty contest” was originally suggested by Maynard Keynes – referring himself to a special kind of competition by English newspapers, where the winner must forecast who among a set of girls would be chosen by the other participants for the competition as the one with prettiest face. “It is not a case of choosing those which, to the best of one’s judgment, are really the prettiest nor even those which average opinion genuinely thinks the prettiest. We have reached the third degree, where we devote our intelligences to anticipating what average opinion expects the average opinion to be” (Keynes 1936, *General Theory of Employment Interest and Money,* p. 140).

Table B1. Selected Projects for the Main Experiment

Market Session	Project Name	(Expected) Returns	Risk (Lottery)		Horizontal-attributes-information				
					Project's Characteristics		Designer's Characteristics		
			Amount	Prob.	Sponsor	Rating	Exp.	Education	Sex
First	Yellow	400	572	0.7	Bank	4	15	Economics	M
	Red	400	1000	0.4	Insurance	4	8	Sociology	F
	Blue	400	1000	0.4	University	2	5	Law	F
Second	Yellow	500	1000	0.5	University	9	6	Law	F
	Red	500	1250	0.4	Bank	2	20	Economics	M
	Blue	500	834	0.6	Insurance	5	2	Engineering	M
Third	Yellow	500	1000	0.5	University	9	6	Law	F
	Red	500	1000	0.5	Curia	6	8	Law	F
	Blue	500	834	0.6	University	3	3	Engineering	M
	Green	500	1250	0.4	Bank	2	20	Economics	M
	Purple	500	834	0.6	Insurance	5	2	Engineering	M
	Orange	500	715	0.7	District	9	5	Economics	M

Notes: The table shows the characteristics of the projects available in each market session. We used the same set of projects in every study and in every treatment. Specifically, in the Baseline treatment, we provided only the name and returns of the project; in the Info treatment, we provided the name, returns, and horizontal-attributes-information related to the project; in the Risk treatment, we provided only the name and the lottery linked to the project; and in the Combined treatment, we provided the name, lottery, and horizontal-attributes-information related to the project.
