# Supplementary material for: Ignorance is bliss? Information and risk on crowdfunding platforms
Source: PLoS One. 2023 Jun 16;18(6):e0286876. doi: 10.1371/journal.pone.0286876 (PMC10275436; doi:10.1371/journal.pone.0286876)
Supplement: S3 Appendix — (PDF) [file pone.0286876.s003.pdf]

S3 Appendix C. Results of Mann–Whitney tests

Table C1. Average amount invested in the winning project in each treatment, for each study.

	Amount invested		
	Study 1	Study 2	Study 3
BASE	137.50 (92%)	134.47 (90%)	131.22 (87%)
INFO	143.75 (96%)	136.65 (91%)	141.28 (94%)
RISK	120.83 (81%)	112.22 (75%)	119.58 (80%)
COMB	114.58 (76%)	124.39 (83%)	103.25 (69%)
	Difference		
	Study 1	Study 2	Study 3
INFO-BASE	6.25 (p=0.3034)	2.18 (p=0.5495)	10.06** (p=0.0369)
RISK-BASE	-16.67* (p=0.0547)	-22.25*** (p=0.0001)	-11.64** (p=0.0333)
COMB-BASE	-22.92** (p=0.0127)	-10.08*** (p=0.0001)	-27.97*** (p=0.0000)
RISK-INFO	-22.92*** (p=0.0046)	-24.43*** (p=0.0002)	-21.69*** (p=0.0000)
COMB-INFO	-29.17*** (p=0.0008)	-12.26*** (p=0.0003)	-38.03*** (p=0.0000)
COMB-RISK	-6.25 (p=0.5444)	12.17 (p=0.4409)	-16.33** (p=0.0206)

Notes: At the top, the table reports average contributions to the winning project overall and by study. At the bottom, the table shows the significance levels from a two-sided Mann–Whitney rank-sum test for the null hypothesis that the mean contributions to the winning project in the two considered treatments are the same. Aggregate data over the three market sessions were considered. Pvalues between parentheses. N = 72 for each study.
* p < 0.1, ** p < 0.05, *** p < 0.01.

Table C2. Average contributions to the winning project over the three market sessions for each study.

	Amount Invested								
	Study 1			Study 2			Study 3		
	MS1	MS2	MS3	MS1	MS2	MS3	MS1	MS2	MS3
BASE	131.25	143.75	137.5	125.25	148.75	129.42	115.29	135.37	143
INFO	143.75	143.75	143.75	127.17	143	139.79	132.79	148.125	142.92
RISK	137.5	118.75	106.25	98.67	124.17	113.84	138.92	116.46	103.37
COMB	143.75	112.5	87.5	115.33	127.83	130	83.125	125.75	100.87
	Difference								
	Study 1			Study 2			Study 3		
	MS1	MS2	MS3	MS1	MS2	MS3	MS1	MS2	MS3
INFO-BASE	12.5 (p=0.3014)	0 (p=1)	6.25 (p=0.5552)	1.92 (p=0.8187)	-5.75** (p=0.0438)	10.37 (p=0.45)	17.5 (p=0.1274)	12.755* (p=0.0564)	-0.08 (p=0.7369)
RISK-BASE	6.25 (p=0.6401)	-25* (p=0.0841)	-31.25* (p=0.0673)	-26.58** (p=0.0152)	-24.58*** (p=0.0009)	-15.58 (p=0.1375)	23.63** (p=0.0426)	-18.91** (p=0.0403)	-39.63*** (p=0.0004)
COMB-BASE	12.5 (p=0.3014)	-31.25** (p=0.043)	-50*** (p=0.0083)	-9.92* (p=0.0655)	-20.92*** (p=0.0001)	-0.58 (p=0.1726)	-32.17** (p=0.0134)	-9.62* (p=0.0757)	-42.13*** (p=0.0006)
RISK-INFO	-6.25 (p=0.5552)	-25* (p=0.0841)	-37.5** (p=0.0215)	-28.5** (p=0.016)	-18.83* (p=0.0802)	-25.95** (p=0.0215)	6.13 (p=0.6160)	-31.67*** (p=0.0001)	-39.55*** (p=0.0001)
COMB-INFO	0 (p=1)	-31.25** (p=0.043)	-56.25*** (p=0.0022)	-11.84* (p=0.0963)	-15.17** (p=0.0186)	-9.79** (p=0.0202)	-49.66*** (p=0.0001)	-22.37*** (p=0.0002)	-42.05*** (p=0.0003)
COMB-RISK	6.25 (p=0.5552)	-6.25 (p=0.734)	-18.75 (p=0.3703)	16.66 (p=0.2894)	3.67 (p=0.7573)	16.16 (p=0.6085)	-55.795*** (p=0.0000)	9.29 (p=0.4702)	-2.5 (p=0.9322)

Notes: At the top, the table reports average contributions to the winning project for each market session of each treatment of each study. At the bottom, the table shows the significance levels from a two-sided Mann–Whitney test for the null hypothesis that the mean contributions in the two considered treatments are the same. P-values between parentheses. N = 24 for each market session.
* p < 0.1, ** p < 0.05, *** p < 0.01.
