# Supplementary material for: Ignorance is bliss? Information and risk on crowdfunding platforms
Source: PLoS One. 2023 Jun 16;18(6):e0286876. doi: 10.1371/journal.pone.0286876 (PMC10275436; doi:10.1371/journal.pone.0286876)
Supplement: S4 Appendix — (PDF) [file pone.0286876.s004.pdf]

S4 Appendix D. Examples of choice windows in the Baseline, Info, and Risk Treatments

Figure D1. Choice Window: Baseline Treatment

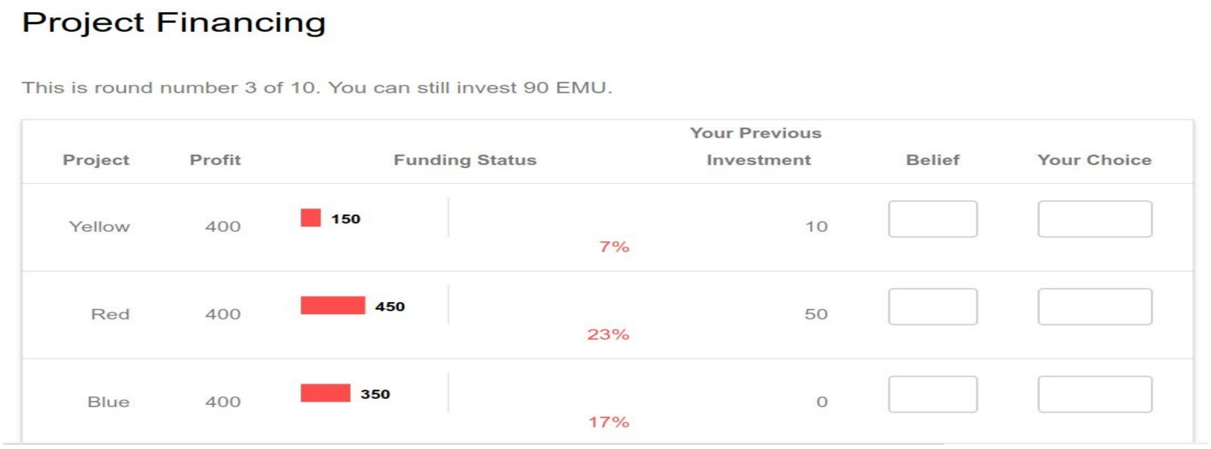

Notes: In the Baseline, participants could only see the name (first column) and the return (second column) of the projects. In each period after the first one the "Funding status" of each project (third column) was updated with the amount of contributions each project was able to collect up to that period. This information was given graphically, with a bar that became green when the threshold was met, with a number indicating the total amount of funding, and with the percentage of collected funding over the total amount needed to fund a project. Participants were also informed over their previous cumulative investment on each of the available projects (column "Your investment"). In the last column of the Table ("Your choice"), participants had to indicate the investment decision for each project, while in the column "Beliefs" they were required to indicate the amount they thought each project would collect in the current round.

Figure D2. Choice Window: Info Treatment

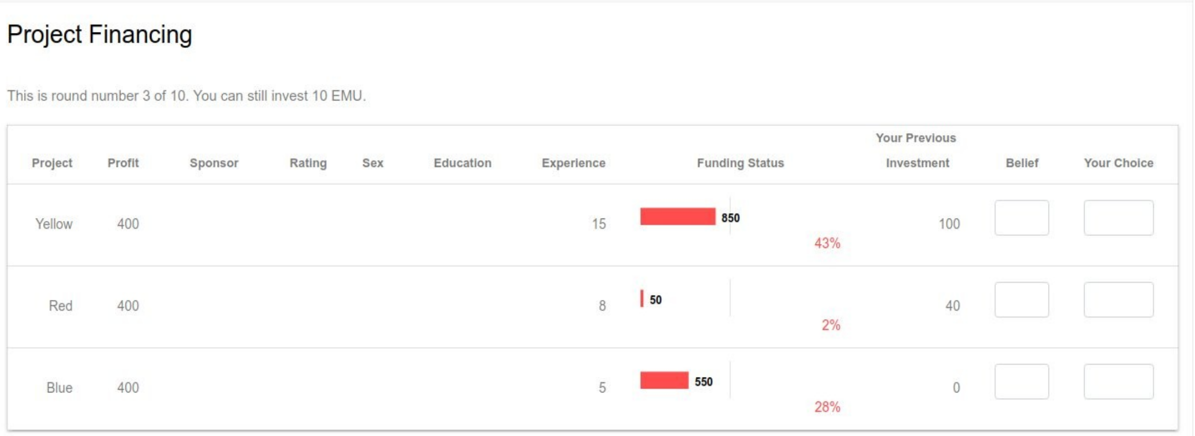

Notes: In the Info treatment we added to the description of the projects the five columns ("Sponsor", "Rating", "Sex", "Education" and "Experience") with the content of the cheap information relative to each project. Participants could view the values of one element of the cheap information at a time by clicking on the corresponding name – the values were visible until he would click to view a different one. In the example, "Experience" has been clicked, showing the corresponding values for each project.

Figure D3. Choice Window: Risk Treatment

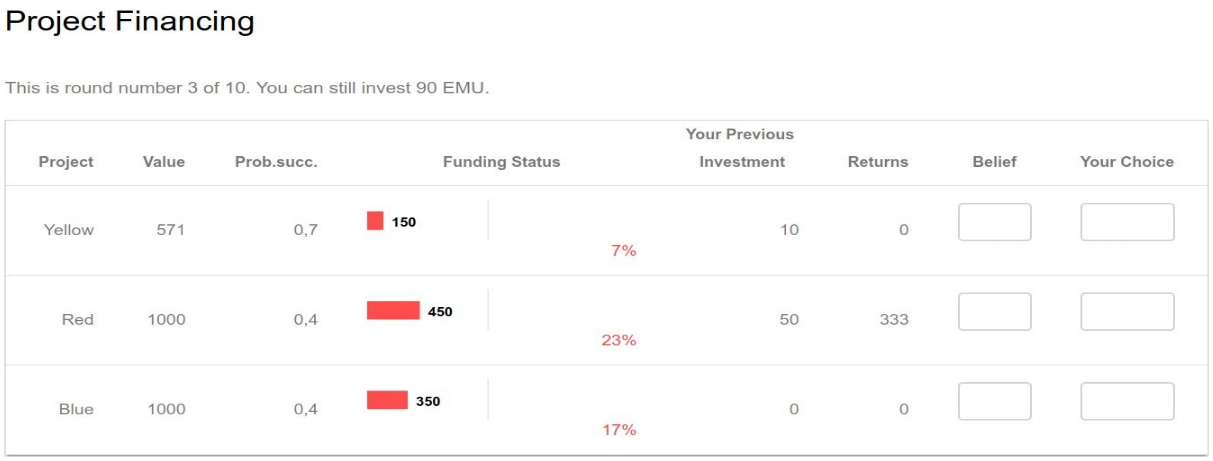

Project Financing

This is round number 3 of 10. You can still invest 90 EMU.

Project	Value	Prob.succ.	Funding Status		Your Previous Investment	Returns	Belief	Your Choice
Yellow	571	0,7	150	7%	10	0		
Red	1000	0,4	450	23%	50	333		
Blue	1000	0,4	350	17%	0	0		

Notes: In the Risk treatment participants could see only the name of the project (column "Project") and the values of the lottery associated with each project: the return in case the lottery is successful ("Value") and the probability of success of the lottery ("Prob.succ."). Like in the other treatments, participants could see the funding status of each project and their personal cumulative investment in each project. We also added a column "Returns", that indicates the participant's return from each project, which was automatically updated in every period, depending on the participant's cumulative investment. In the example, the participant invested 10 EMU in the Yellow project, which is not enough to get a positive return (the corresponding return is zero), while he invested 50 EMU in the Red project, which would ensure him the 1/3 of the value of the project (the corresponding return is 1/3 of 1000 EMU, i.e. 333 EMU), in the case the project reaches the threshold and the corresponding lottery is successful.
