# Supplementary material for: Ignorance is bliss? Information and risk on crowdfunding platforms
Source: PLoS One. 2023 Jun 16;18(6):e0286876. doi: 10.1371/journal.pone.0286876 (PMC10275436; doi:10.1371/journal.pone.0286876)
Supplement: S5 Appendix — (PDF) [file pone.0286876.s005.pdf]

S5 Appendix E. Instructions (Combined Treatment)

Welcome! We thank you for accepting to participate in this experiment, which will allow you to earn some money. You have already earned 2.5 Euro to arrive on time. During the experiment you will not be able to communicate with the other participants. If something in the instructions is not clear to you, please raise your hand and ask for information directly from the people conducting the experiment. The payment you will receive at the end of the experiment will be determined by the choices you make and by the choices that will be made by other participants, according to the methods that will be explained below. During the experiment, the amounts are expressed in EMU (Experimental Monetary Units), where 50 EMU = 1 Euro, or 1 EMU = 0.02 Euro. This experiment is divided into two parts. The instructions for the first part of the experiment are given below. You will be given instructions on the second part later. During the experiment your choices will remain anonymous.

First part – Instructions

The aim of this experiment is to simulate a project market. In a previous experiment, we asked other participants, called designers, to design a set of projects. Each designer had the opportunity to choose the characteristics of a project, in order to make it attractive to the largest possible number of lenders. You and the other participants of today can finance one of these projects, which will be indicated by different colors (blue, pink, white, ect). In order to be financed, a project must reach a minimum funding threshold, which is the same for all the projects. If a project exceeds the minimum funding threshold, it might generate profits for all the lenders who have invested in that project. The profits of a financed project will depend on the result of a lottery, according to the procedures that will be described later. Each participant will receive one share at the beginning of the experiment, valued at 150 EMU, and can choose to hold it, or use it to invest in only one project. The experiment consists of 10 rounds. In each round after the first, you can see the level of funding that each project has gotten until that round, and if you have not already invested your share, you can decide whether to invest it in a project or to wait, but you cannot get back a share you have already invested. Since the minimum funding threshold for a project is 13 shares, at least 13 lenders will be needed to reach the threshold for the realization of the project, and only one project can be financed.

If a project fails to reach the minimum funding threshold, any lenders of that project will be partially reimbursed of the invested portion, according to the procedures that will be described later.

The projects

The Projects are characterized by the following elements:

- Value of the project
- Probability of success
- Type of institution that sponsors the project
- Reputation of the project

The designers are instead characterized by the following characteristics:

- Education
- Experience

These characteristics will be summarized in a table like the following:

Project	Value	Prob.succ.	Sponsor	Rating	Sex	Education	Experience
Yellow	1000	0.5	University	8	F	Law	6

Figure E1: Example of a project

To view each characteristic, you will have to click on the corresponding name, and the respective values for each project will appear. You will be able to view one characteristic at a time, but you can click on each name as often as you wish. Moreover, by passing the mouse over the name of each characteristic, a window will be opened with a brief explanation of the variable.
Below you will find the explanation of each characteristic of the projects:

Value of the project
As already mentioned, if a project reaches the funding threshold, your profit will depend on the result of a lottery. A lottery is made of a given probability of success and a corresponding profit. The first characteristic that you will find in the table is the value of the project, that indicates the profit generated by the project in the case of success, that is, if the project reaches the minimum funding threshold and the lottery is successful.

Probability of success
The second characteristic is the risk of the project, that is, the probability with which the funded project is able to generate profits. This variable is related to the value of the project, since the higher is the value of the project, the lower is the probability to obtain the corresponding profit. For example, to a probability of 10% corresponds a profit of 10000 EMU, while to a probability of 90% corresponds a profit of 1111 EMU.

Type of institution that sponsors the project
The third characteristic of the project is the institutional sponsor. The designers of the projects have in fact chosen the sponsor from a list of various proposed institutions that they have thought is the sponsor that will make the project most attractive for lenders.

Reputation of the project
The fourth characteristic of the project indicates the reputation of the project among the designers. After having defined the various projects, the designers were asked to vote for the projects based on their perception of which project will be the most likely to reach the funding threshold. The third characteristic indicates how many votes each project received during this evaluation.

Below we provide the explanation of each characteristic of the designer:

Designer education
This characteristic indicates the education of the designer of the project, that is, in which degree programme is the designer enrolled in.

Designer experience
This characteristic indicates the experience of the designer accumulated by having participated in experiments in the past. The experience is indicated by the number of experiments in which the designer took part until now.

Designer Gender

Your choice
In each round, you can decide whether to invest your share in a project or to wait, by selecting the appropriate option: Once your share has been invested, it is not be possible to get it back or to change project in the following rounds. In each round you will also have the following pieces of information:

Collected shares
It indicates the total shares that the project collected until that round. Both the total number of shares collected and the percentage of funding will be indicated. Furthermore, bar charts are used to make the comparison between the funding levels of the different projects more intuitively understandable. The bar graph will be red if the corresponding project has not yet reached the minimum funding threshold, and, once the threshold is reached, it will turn green. It is possible to participate in the financing of a project, even when this has already reached the minimum funding threshold. When making your choice, before clicking on a characteristic, you will visualize the following window:

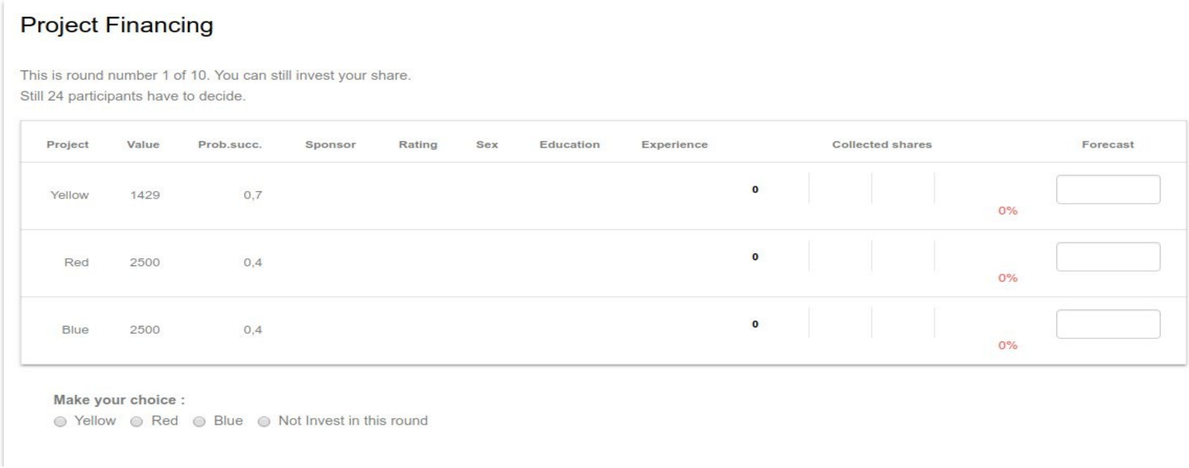

Figure E2: Choice Window (Combined-treatment)

In each round you will also be informed of how many participants have not yet chosen to invest. In the last column of the table, called forecasts, you will have to indicate how many participants, among those who have not yet chosen to invest, are choosing each project during the current round.

Experimental Procedure:
As anticipated, the experiment consists of 10 rounds. In each round you can indicate whether to invest your share in a project, or to wait. You can invest in a project even if this has already reached the funding threshold. You can decide not to invest in any project and to keep your share. In each round following the first, you will have information on the level of financing of the projects. Remember that only one project can be financed, because the sum of shares available to the lenders, is such that only one project at most can be financed. It is important to note that once you have invested your share in a project, it will no longer be possible to change your decision, i.e. you will not be able to remove your share and transfer it to another project, nor will you be able to get back your share. Remember that, in each round of the experiment, you will also need to indicate the number of subjects you believe will invest in each project during that round.

Profits calculation
After the last round, profits will be calculated. If a project has reached the minimum funding threshold, it can generate profits, according to the result of the corresponding lottery. In particular:
• If you have invested in a project, that reaches the funding threshold, and won the lottery, you will receive the value of the project. For example, if the project value is 450 EMU, you will receive 450 EMU.
• If you have invested in a project, that reaches the funding threshold, and didn’t win the lottery, you will lose your share and your profit will be 0 EMU.
• If you have invested in a project, and the project does not reach the funding threshold, you will receive 50 EMU, as a partial refund of the amount invested.

• If you have not invested in any project, you will keep your initial share, i.e. 150 EMU.

Sessions

During the experiment you will participate in three sessions, i.e. three markets of projects. In every session, the above-explained procedure is repeated. This means that each session will last 10 rounds, and in each session you will receive a share that you can use to finance a project from those available in the session. Within each session, projects will have the same value and the same funding threshold. The value of projects can change between one session and another.

Payments

Each participant will receive 2.5 Euro for participating in the experiment. For this part of the experiment, the gain will be calculated as follows: at the end of the last session, one of the three sessions (markets) will be randomly drawn, and your earnings will be equal to your profit in the selected session. within the selected session, one round will be randomly drawn and the number of participants who have invested in each project during that round will be calculated. The participant who indicated the number of subjects that is closest to the true number, will receive 100 EMU. If there are any equal-merit, the winner will be randomly picked among them.

Control questions

1. There are 10 rounds to finance a project within each session. True or false?
2. In the randomly selected session the White project was financed, worth 450 EMU, and it didn't win the lottery. You have invested in the White project. Your profit is of 0 EMU. True or false?
3. Suppose that you invest your share on a project in the third round. At the seventh round you decide to remove your share from that project, and invest it on another project. Is it possible?
4. Your profit is given by the sum of the profits obtained in each session. True or false?
5. In the randomly selected session the White project was financed, worth 450 EMU, and it won the lottery. You have invested in the White project. What is your profit?
6. In the randomly selected session the White project was financed, worth 450 EMU. You have invested in the Black project. What is your profit?
7. In the randomly selected session the White project was financed, worth 450 EMU. You have decided to keep your share and to not invest it. What is your profit?
8. At each round you will have to indicate how many players have already invested in each project. True or false?

Second part - Instructions

On your screen you will see 100 boxes. In one of them, there is a bomb; each of the other 99 boxes contains 2 EMU. You do not know where the bomb is, but you know that it might be in any of the 100 boxes with the same probability. Your task is to select all the boxes you want. You will earn 2 EMU for each box that you collect without the bomb. To select a box, you can simply click on it. Selection does not imply that it will immediately open; instead, you will discover the actual contents (EMU or the bomb) of your boxes only at the end of the experiment. If you select the box containing the bomb, then everything you have collected is destroyed and you will earn 0 EMU for this second phase of the experiment. After collecting all the boxes you like, select the STOP button. This ends the second phase of the experiment. The earnings (in EMU) you acquire in this second phase of the experiment will be added to the earnings (in EMU) you obtained during the first phase of the experiment.
